# Unified Saliency Detection Model Using Color and Texture Features

**DOI:** 10.1371/journal.pone.0149328

**Published:** 2016-02-18

**Authors:** Libo Zhang, Lin Yang, Tiejian Luo

**Affiliations:** School of Computer and Control, University of Chinese Academy of Sciences, Beijing, China; College of Mechatronics and Automation, National University of Defense Technology, CHINA

## Abstract

Saliency detection attracted attention of many researchers and had become a very active area of research. Recently, many saliency detection models have been proposed and achieved excellent performance in various fields. However, most of these models only consider low-level features. This paper proposes a novel saliency detection model using both color and texture features and incorporating higher-level priors. The SLIC superpixel algorithm is applied to form an over-segmentation of the image. Color saliency map and texture saliency map are calculated based on the region contrast method and adaptive weight. Higher-level priors including location prior and color prior are incorporated into the model to achieve a better performance and full resolution saliency map is obtained by using the up-sampling method. Experimental results on three datasets demonstrate that the proposed saliency detection model outperforms the state-of-the-art models.

## Introduction

Visual attention is a significant mechanism of the human visual system (HVS). It allows humans to select the most relevant information on visual information from the environment. Visual attention is modeled as saliency detection in computer vision. Saliency detection is to find conspicuous areas and regions of input image then output a gray scale image, which is called saliency map. In recent years, saliency detection has drawn a lot of interest in computer vision. It provides fast solutions to several complex processes and has attracted a lot of attention from numerous universities and research institutes. In the past decades many saliency models have been proposed and widely exploited in image segmentation [1,2], object recognition [3,4,5], image retrieval [6], image resizing [7], image/video compression [8,9] and image/video quality assessment [10,11].

Visual psychology studies show that the human visual system mechanism is driven by two factors: (i) a bottom-up component, which is fast, data driven, and pre attentive, (ii) a top-down component, which is slow, goal driven, and attentive. Many bottom-up saliency detection methods have been proposed. This method can provide a lot of useful information and many successful models have been proposed that have made great achievements. But this kind of method is only based on low-level information such as color, intensity and orientation. And it doesn’t consider prior knowledge about the input image. However, the high-level factors are necessary for HVS and saliency detection. Top-down based saliency detection is the latest progress in visual attention area. The appropriate and effective utilization of high-level information can improve the performance of current bottom-up based saliency detection models. While most studies on top-down based saliency detection are still at descriptive and qualitative level, few completely implemented computational models are available [12]. Inspired by the works of fusing bottom-up and top-down factors [12,13,14], this paper proposes a saliency detection model which fuses bottom-up features with adaptive weight and incorporate higher-level priors to the model.

The remainder of this paper is organized as follows. Section 2 introduces related work briefly. Section 3 describes the proposed salient region detection model. In Section 4, we evaluate the performance of the proposed model by comparing with state-of-the-art methods. We conclude the paper in Section 5.

## Related Work

IItti et al. [15] used center-surrounded differences across multi-scale image features to define saliency of image. Tie Liu et al. [16] represented the salient object as a binary mask and formulated the salient object detection problem as a binary labeling task. Then they extended their method to the sequential image case by exploring the extra temporal information. Zhixiang Ren et al. [17] proposed a region-based saliency detection method and applied the achieved saliency map in object recognition task. M. M. Cheng et al. [18] presented a regional contrast based salient object detection algorithm that evaluated global contrast differences and spatial weighted coherence scores. They used the saliency maps to accomplish unsupervised salient object segmentation. Yonghong Tian et al. [19] learned complementary saliency priors for object segmentation, which was formulated as binary pixel labeling problem by learning two complementary saliency maps that most likely reveal foreground and background respectively. Achanta et al. [20] introduced a frequency tuned saliency detection method. Using the color differences from the average image color to define pixel saliency. Yanfei Ren [21] reported a saliency map generation method that extracting texture feature and combining a new feature fusion strategy. F. Perazzi et al. [22] decomposed the input image into perceptually homogeneous elements and estimated saliency based on uniqueness and spatial distribution of those elements. Chen Xia et al. [23] proposed a nonlocal reconstruction-based saliency model, their model focused more on the original image’s sparsity and uniqueness. Li Zhou et al. [24] proposed a bottom-up saliency detection model. Their model integrated compactness and local contrast cues using diffusion process to produce a pixel-accurate saliency map. Lei Zhu et al. [25] computed both local center-surround contrast and global saliency between multisize superpixels, showed that multi-scale scheme can improve the performance of local saliency approaches.

The idea of integrating top-down factor to saliency estimation was first proposed by Itti and Koch [26] in 2001. They found that there was a link between visual attention and eye movement. In recent years, many top-down saliency models had been presented. Xiaohui Shen et al. [13] incorporated low-level features with higher-level guidance to detect salient objects. In their model, original image was represented as a low-rank matrix plus sparse noises, which were used to indicate non-salient regions and salient regions respectively. Zhenzhong Chen et al. [27] considered the high-level cue imposed by the photographer and integrated the defocus map of the image with low-level features. Tao Deng [28] introduced a top-down based saliency model regarding vanishing points of road as top-down guidance and applying it in traffic driving environment. Xiaoguang Cui et al. [29] proposed a top-down visual saliency detection method to process optical satellite images, which measured the local similarity of a pixel to its neighbor pixels.

Yin Li et.al [30] found that the existing salient object benchmarks have serious design flaws because of overemphasizing the stereotypical concepts of saliency, which called the dataset design bias. Center bias was the tendency of subjects looking at the screen center more often [31]. In their view, the most significant bias was center bias. Yin Li et.al had done an excellent work that make researchers think more about dataset design problem, and that might open up a new filed in saliency detection. However, their conclusions had not been widely accepted. Existing benchmarks are still used by many recent published work [32,33,18]. In this paper, benchmarks including THUS10000, MSRA1000, and DB-Bruce are used to test the performance of our proposed model.

## The Proposed Model

**Fig 1:**
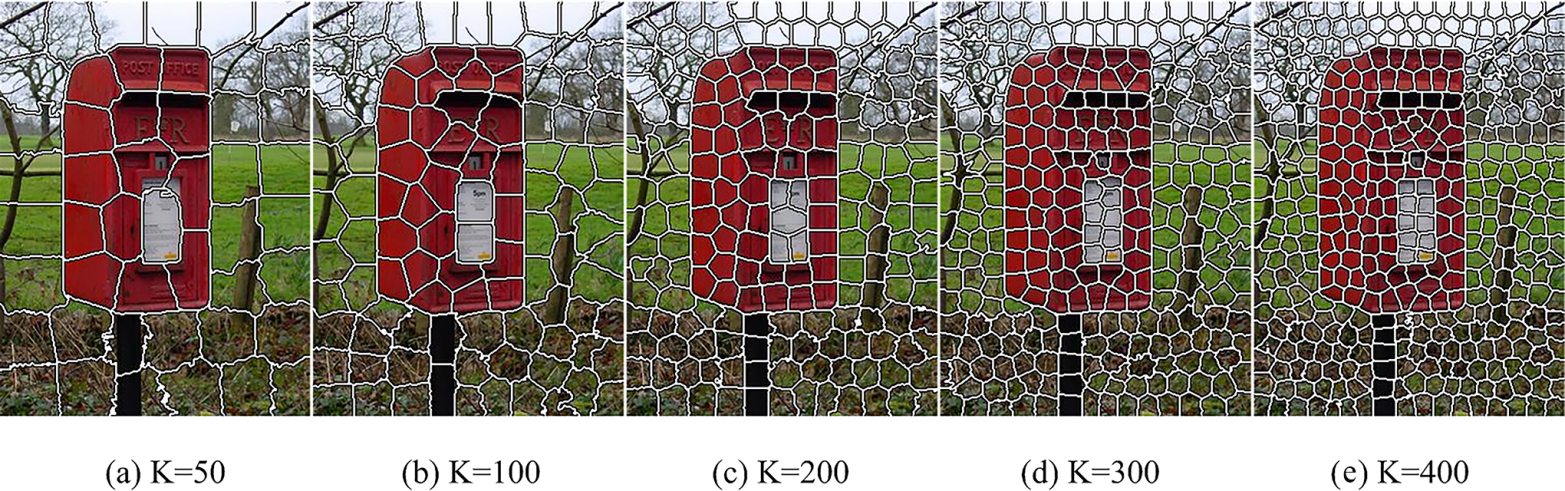
Result of SLIC superpixels oversegmentation with different number of superpixels.

**Fig 2:**
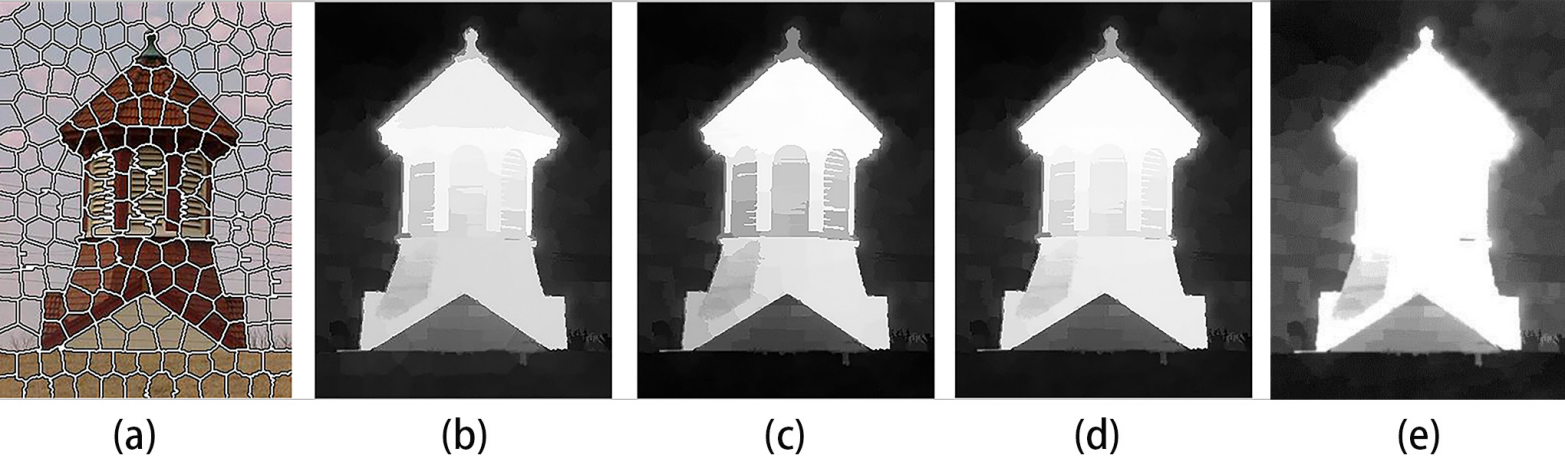
Illustration of the main phases of the proposed model. (a) Oversegmentation of input image; (b) Color saliency map; (c) Texture saliency map; (d) Fuse color and texture saliency map; (e) Final saliency map that incorporate high-level priors.

Approach to our proposed model is divided into five main parts: colors saliency map, texture saliency map, saliency map fusion, integration of high-level prior and full resolution saliency map. At first, Input image is segmented into N sub-regions by utilizing SLIC superpixels oversegmentation algorithm [34]. The SLIC superpixels algorithm uses K-means clustering approach to generate superpixels in CIELAB space, which is fast, memory efficient and adhere to boundaries. Fig 1 shows the sample result of SLIC superpixels oversegmentation. Then we calculate Color saliency map and texture saliency map based on region contrast method and fuse the two maps by adaptive weight. Next, higher-level priors including color prior and location prior are incorporated into the model to get a better performance. Lastly, we use the up-sampling method to get full resolution saliency map. The main process of the proposed model is illustrated in Fig 2.

### Color Saliency Map

For a sub-region *r*_*i*_, we compute color feature map by the following formula:

$$S_{col}(r_{i})=\sum_{i\neq k}\omega (r_{i},r_{k}){\left\|I_{i}-I_{k}\right\|}^{2}$$ (1)

$$\omega (r_{i},r_{k})=\text{exp}(-D_{e}\left(r_{i},r_{k}\right))/C_{i}$$ (2)

*I*_*i*_ is the mean color of all pixels in sub-region *r*_*i*_, *D*_*e*_(*r*_*i*_, *r*_*k*_) is the Euclidean distance between sub-region *r*_*i*_ and *r*_*k*_, *C*_*i*_ is scale factor that make sure ∑_*i* ≠ *k*_ ω(*r*_*i*_,*r*_*k*_) = 1.

### Texture Saliency Map

We calculate the texture saliency map based on the method of [21]. The saliency value of each region is given as follows:

$$S_{tex}(r_{i})=\sum_{k=1}^{N}\omega (r_{i},r_{k})D_{t}(r_{i},r_{k})$$ (3)

*D*_*t*_(*r*_*i*_, *r*_*k*_) refers to the Euclidean distance on texture feature value between the two sub-regions, which is given by:

$$D_{t}(r_{i},r_{k})=\sum_{m=1}^{l_{m}}\sum_{n=1}^{l_{n}}f(t_{i,m})f(t_{k,n})D_{e}(t_{i,m},t_{k,n})$$ (4)

*l*_*m*_ and *l*_*n*_ respectively represent the number of texture type in region *r*_*i*_ and *r*_*k*_, *f*(*t*_*i*,*m*_) is the frequency of the m-th texture feature among all texture features in region *r*_*i*_. The frequency of a texture feature reflect the differences between textures, which can be used to measure the weight of this texture feature.

### Saliency Map Fusion

The color saliency map and texture saliency map are linearly combined with adaptive weights, which are adjusted adaptively to the *DoS* (degree-of-scattering) and eccentricity of each feature map [14]. The final saliency value S(*r*_*i*_) of region *r*_*i*_ is given by:

$$S(r_{i})=\alpha S_{col}(r_{i})+\beta S_{tex}(r_{i})$$ (5)

*S*_*col*_(*r*_*i*_) and *S*_*tex*_(*r*_*i*_) are color saliency and texture saliency of region *r*_*i*_ respectively. *α* and *β* are the weights of color saliency and texture saliency respectively.

The *DoS* of saliency map can be used to determine the weighting parameters because the salient region are small and dense in general. The *DoS* of saliency map is defined as the variance of spatial distances between the centroid of saliency map and all sub-regions. The following three steps are used to calculate *DoS*:

Firstly, the centroids of color saliency map *H*^*col*^ and texture saliency map *H*^*tex*^ are calculated with the method proposed in [35]. The centroid eccentricities of saliency maps are used to select the centroid of color saliency map or texture saliency map as the final centroid, which are defined as:

$$E^{O}=\frac{\left\|H^{O}-\bar{H}\right\|}{\left\|H^{col}-\bar{H}\right\|+\left\|H^{tex}-\bar{H}\right\|},\;O\in \{col,tex\}$$ (6)

$\bar{H}$ is the mean position of the centroid of color saliency map and texture saliency map. The centroid of the saliency map with a lower eccentricity is the final centroid because its reliability is better. The final centroid *H* is determined by the following formula:

$$H=\{\begin{array}{c} H^{col},\;E^{col}\leq E^{tex}\\ H^{tex},\;E^{col}>E^{tex} \end{array}$$ (7)

Secondly, we select salient regions by an adaptive threshold *T*, and the Threshold *T* is defined as the mean saliency of saliency map:

$$T=\frac{\sum_{i=1}^{N}S_{i}}{N}$$ (8)

*S*_*i*_ is the saliency value of region *r*_*i*_, *N* is the number of sub-regions. The regions whose saliency value meet *S*_*i*_ > *T* is defined as salient regions. Then we compute the average distance between the final centroid and all salient regions which is given as follow:

$$\bar{d}=\frac{\sum_{n=1}^{S_{N}}\left\|p_{n}-H\right\|}{S_{N}}$$ (9)

*S*_*N*_ is the number of salient regions, *p*_*n*_ is the position of salient region *n*, *n* ∈ [1, *S*_*N*_].

Finally, the variance of spatial distances between the final centroid H and all salient regions is calculated, which is defined as:

$$D=\frac{\sum_{n=1}^{S_{N}}{\left(\left\|p_{n}-H\right\|-\bar{d}\right)}^{2}}{S_{N}}$$ (10)

We define the *DoS* of saliency map by knowing that a higher variance implies higher *DoS*, and vice-versa. The formula is given as:

$$DoS^{O}=\frac{D^{O}}{D^{col}+D^{tex}}\;,\;O\in \{col,tex\}$$ (11)

As mentioned above, the saliency map with higher *DoS* is assigned with a lower weight. Therefore, we set the *α* and *β* by following formula:

$$\{\begin{array}{c} \alpha =1-DoS^{col}\\ \beta =1-DoS^{tex} \end{array}$$ (12)

### Integration of High-level Prior

Based on human perception, we incorporate location prior and color prior to the saliency detection model.

For location prior, the objects near the image center are more attractive, which has been proven by many eye tracking datasets [36]. The location prior map is Gaussian distributed based on the distance of the sub-regions to the image center, its formula is given as:

$$P_{l}(r_{i})=\text{exp}(-d(r_{i},c)/{\sigma_{1}}^{2})$$ (13)

For color prior, warm colors such as red and yellow are more conspicuous. Similar to [13], we obtain a 2-D histogram distribution *H*(*r*_*i*_) of sub-regions in *nR-nG* color space. Different to [13], we set $nR=\frac{R}{R+G+B},nG=\frac{0.5G}{R+G+B}$. Then the corner regions are regarded as background and its histogram distribution *H*(*B*) is generated as well. We get the values from the two histograms *h*_*i*_ and *h*_*B*_. And color prior is given as follow:

$$P_{c}(r_{i})=\text{exp}((h_{i}-h_{B})/\sigma_{2}{}^{2})$$ (14)

Lastly, the above two prior maps are fused into the last saliency map *S*_*i*_, which is assigned as:

$$S_{i}=S(r_{i})•P_{l}(r_{i})•P_{c}(r_{i})$$ (15)

### Full Resolution Saliency Map

To get full resolution saliency map, we assign the saliency value to each pixel by using the up-sampling method proposed in [14] and [35]. The saliency value of a pixel $\bar{S_{j}}$ is a weighted linear combination of the saliency *S*_*j*_ and other sub-regions, which is given as:

$${\bar{S}}_{j}=\sum_{i=1}^{N}\omega_{ji}S_{i}$$ (16)

And the *ω*_*ji*_ is Gaussian weight to ensure the process is both local and color sensitivity. It is defined as below.

$$\omega_{ji}=\frac{1}{\lambda_{j}}\text{exp}(-\frac{1}{2}(a{\left\|c_{j}-c_{i}\right\|}^{2}+b{\left\|p_{j}-p_{i}\right\|}^{2}))$$ (17)

*a*and *b* are parameters that control the sensitivity to color and position. Similar to [14], we set *a* = 1/30 and *b* = 1/30.

## Experiments

In this section, we conduct experiments on three datasets in order to evaluate the performance of the proposed model. We compare our model with these existing works: frequency tuned method (FT) [20], low-rank matrix recovery based method (LR) [13], graph-based method (GB) [37], Itti’s method (IT) [15], region-contrast method (RC) [38], AC [39], context-aware method (CA) [40], spectral residual approach (SR) [41], MSS [42]. The datasets we use are THUS10000(provided by M. M. Cheng [18]), MSRA-1000 (provided by Achanta) and DB-Bruce [43] (**S1 Dataset**).

The saliency map is segmented according to the fixed threshold *T*_*f*_, which ranges from 0 to 255. The regions whose saliency values are higher than *T*_*f*_ are regarded as salient regions. There are 256 binary segmentations by thresholding the saliency map with 256 threshold values. We calculate precision rate by the formula as follows:

$$precision=\frac{tp}{sm}$$ (18)

tp is the number of pixels inside the salient regions of both saliency map and ground truth map, sm is the number of pixels inside the salient regions of saliency map. Recall rate is given as:

$$recall=\frac{tp}{gt}$$ (19)

*gt* is the number of pixels inside the salient regions of ground truth map. Then the F-measure is defined as:

$$F_{m}=\frac{(1+\beta^{2})precision\times recall}{\beta^{2}\times precision+recall}$$ (20)

Similar to [20], we set *β*^2^ = 0.3 to weigh precision over recall.

### Evaluation of Texture, Color, and Final Saliency Map

**Fig 3:**
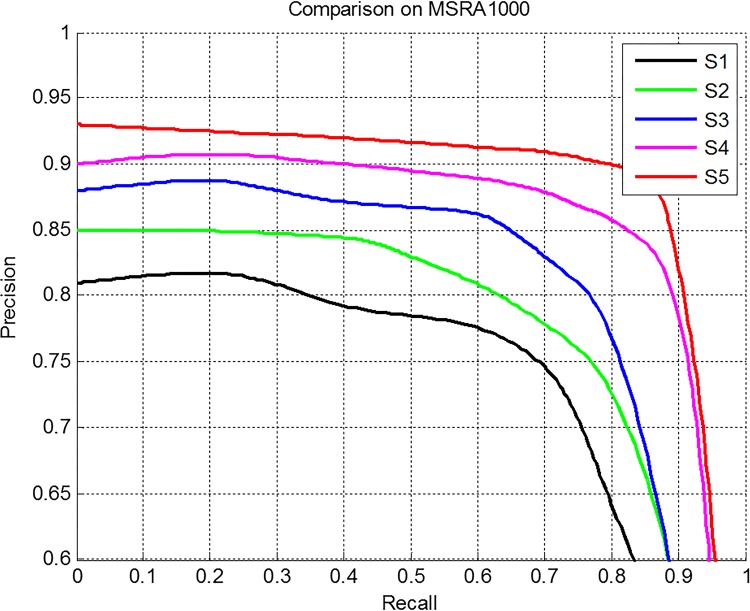
Evaluation of texture (S1), color (S2), fuse texture and color with average weight (S3) and adaptive weight (S4), and final saliency map (S5).

In this section, we evaluate the performance of the texture saliency map, color saliency map, saliency map fusing the texture and color saliency with average weight and adaptive weight respectively and final saliency map on MSRA-1000. Fig 3 shows the average precision recall curves of above five saliency maps. The curves show that the fused saliency map using adaptive weight has better performance than using average weight. And the final saliency map that incorporate high-level prior achieve the best performance among the five saliency maps.

### Comparison on THUS10000

**Fig 4:**
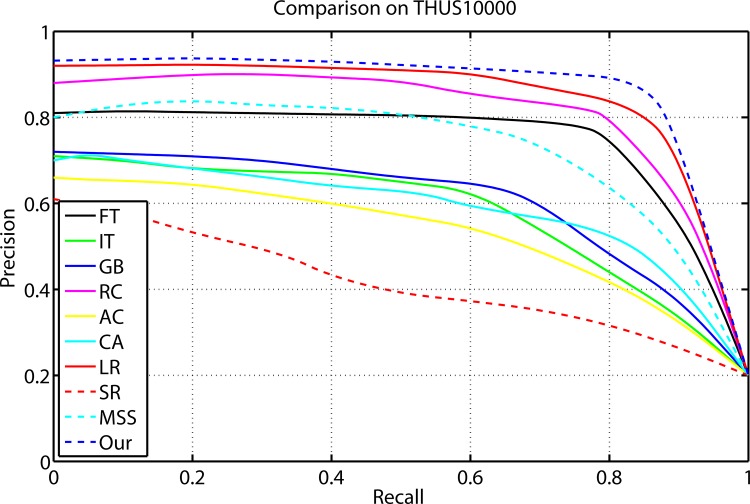
Precision and recall rates of the compared models on THUS10000.

**Fig 5:**
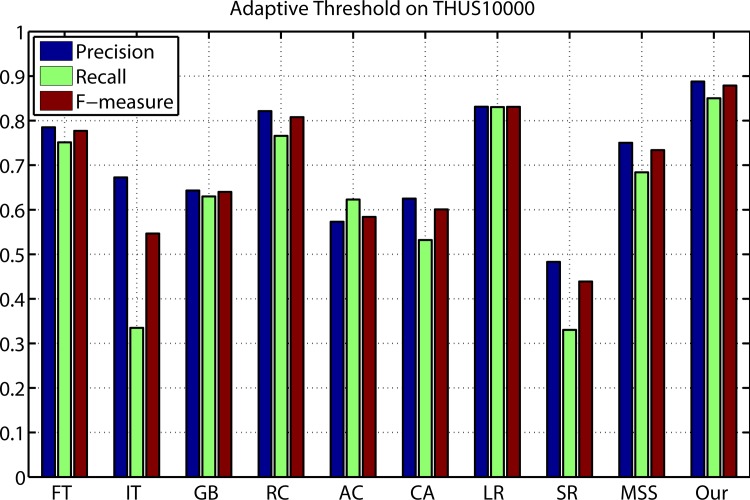
Precision, recall, and F-measure results from adaptive thresholds on THUS10000.

Fig 4 shows the evaluation results of the proposed method compared with nine kinds of different state-of-art saliency detection approaches on THUS10000 data set. The average precision recall curves display that our model outperforms other salient region detection models at every threshold and any recall rate. The average precision, recall, and F-Measure of different methods with an adaptive threshold are shown in Fig 5. The proposed method achieved the highest precision, recall and F-measure. The three benchmarks consistently prove that our method is superior to other nine models.

The proposed method highlighted the salient object regions effectively with well-defined boundaries and suppressed the background regions.

### Comparison on MSRA-1000

**Fig 6:**
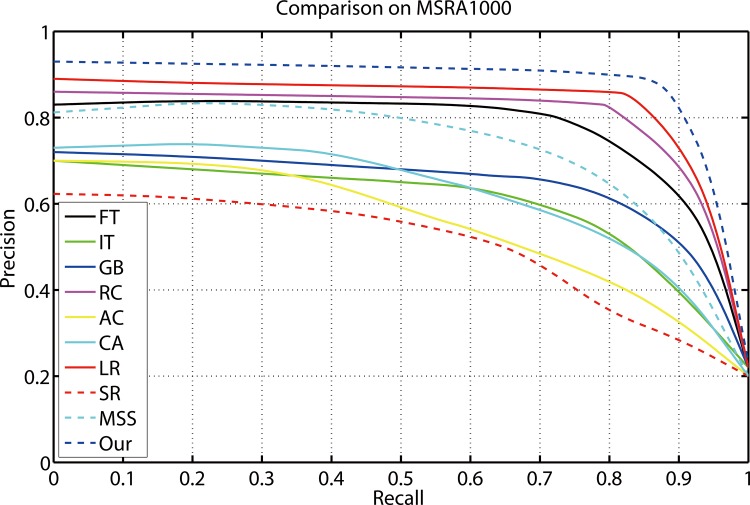
Precision and recall rates of the compared models on MSRA-1000.

Fig 6 shows the evaluation results of the proposed method compared with nine kinds of different state-of-art saliency detection approaches on MSRA-1000 data set. The average precision recall curves display that our model outperforms other salient region detection models at every threshold and any recall rate. The average precision, recall, and F-Measure of different methods with an adaptive threshold are shown in Fig 5. The proposed method achieved the highest precision, recall and F-measure. The three benchmarks consistently prove that our method is superior to other nine models.

### Comparison on DB-Bruce

**Fig 7:**
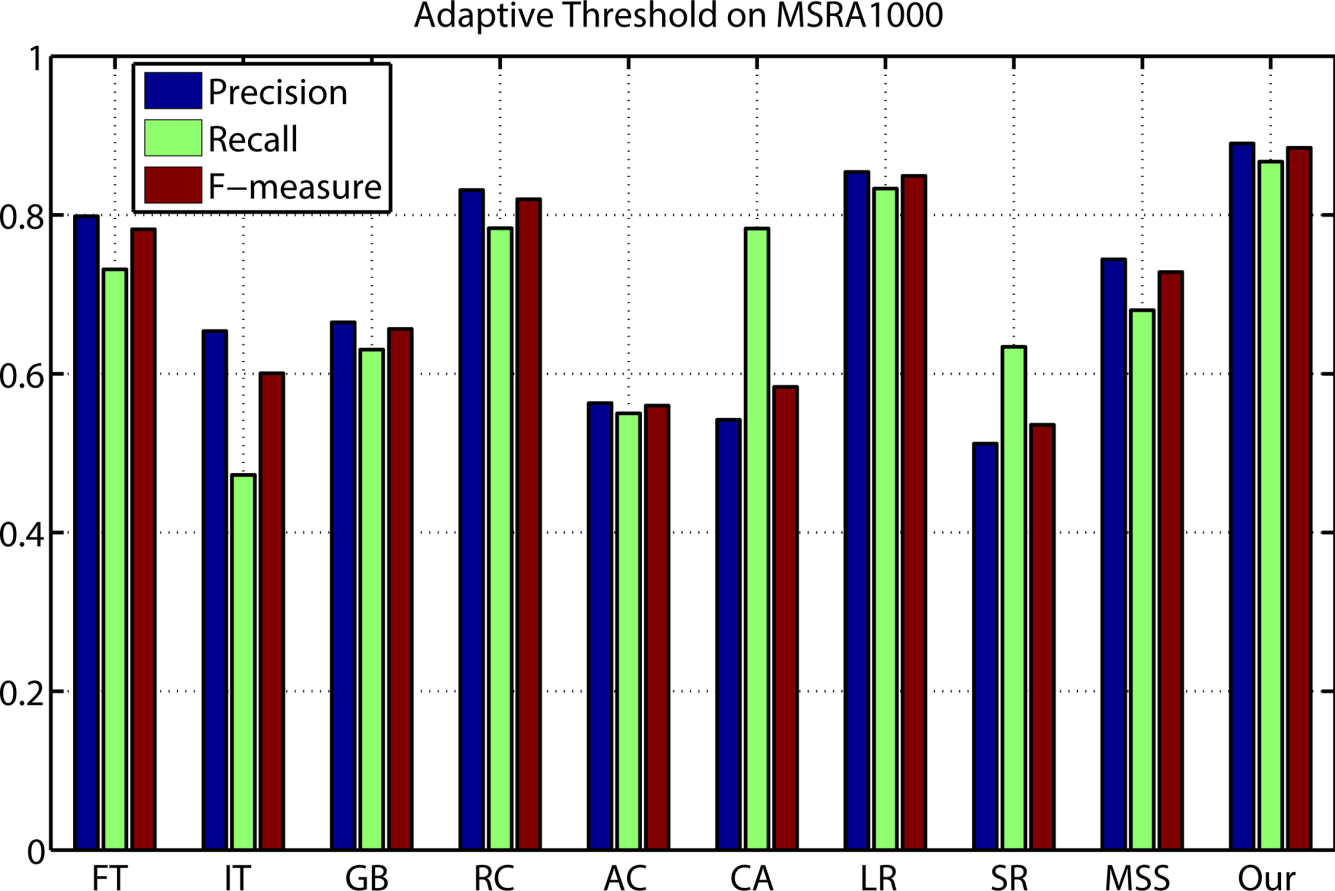
Precision, recall, and F-measure results from adaptive thresholds on MSRA-1000.

**Fig 8:**
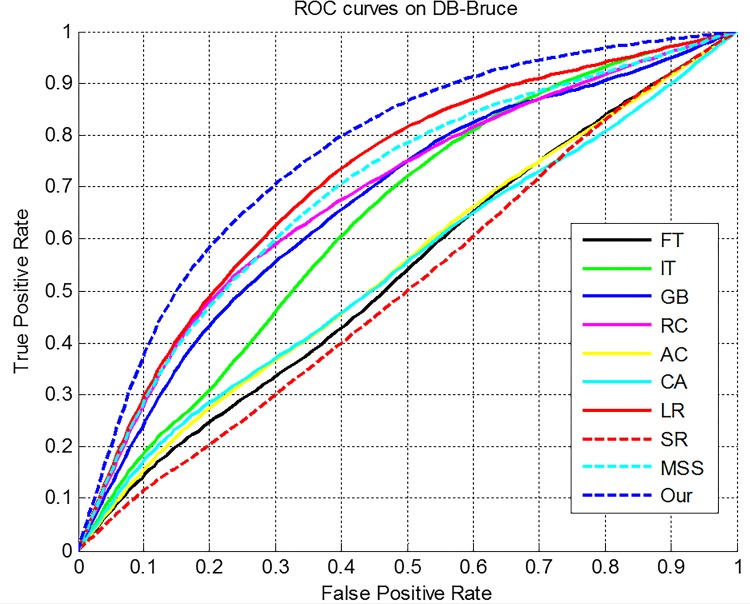
ROC curves of the compared models on DB-Bruce.

DB-Bruce is an eye tracking dataset including 120 images with eye fixation data. We adopt it to evaluate the prediction performance of the proposed model. Here, we use the receiver operating characteristic (ROC) curve and the area under the ROC curve (AUC) [44] to evaluate the performance of saliency detection models. As shown in Fig 7, the ROC curves of our model outperforms other models. Fig 8 provides the comparison results for ROC curves and Table 1. gives the AUC results. As shown in Table 1, the AUC of our method is highest among the compared models.

**Table 1:** Units for the AUC Comparison on DB-BRUCE Database.

Method	FT	IT	GB	RC	AC	CA	LR	SR	MSS	Our
AUC	0.6774	0.7158	0.6934	0.7208	0.6573	0.6809	0.7335	0.5442	0.7208	0.8013

## Conclusion

**Fig 9:**
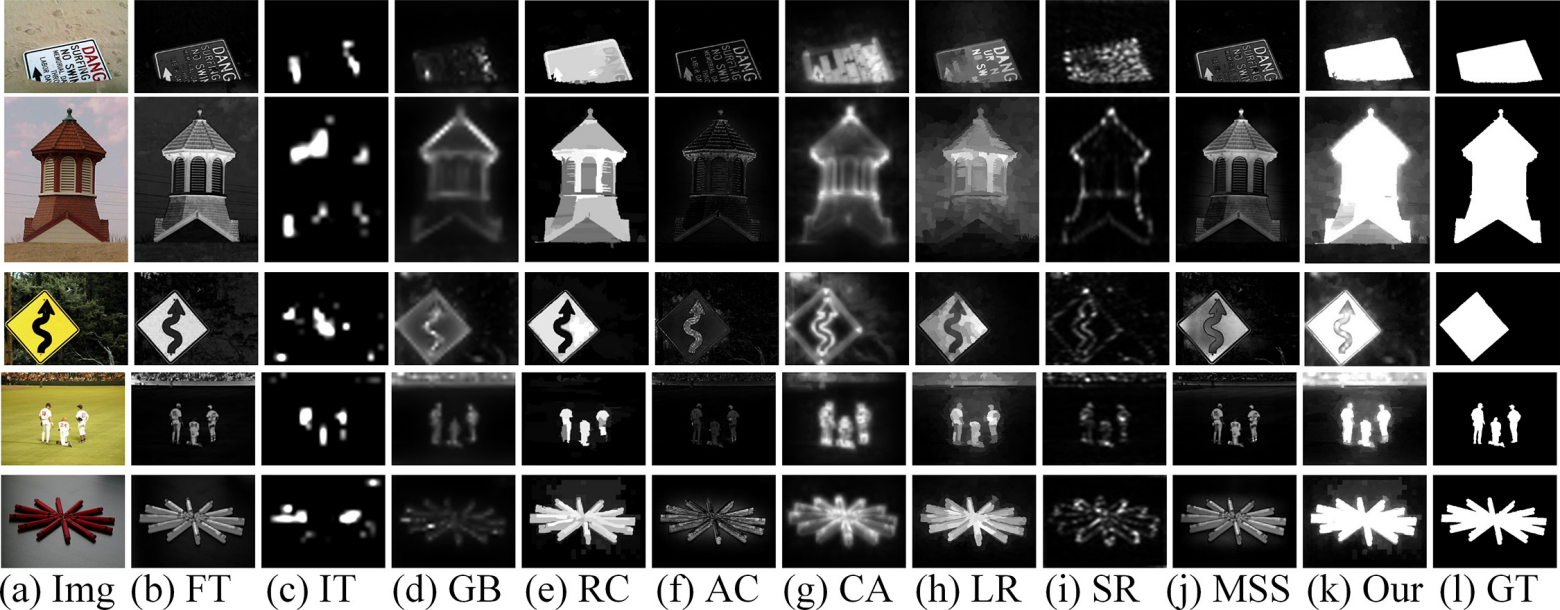
Visual comparison of saliency maps. (a) original images, (b) frequency tuned method (FT) [20], (c) Itti’s mettod (IT) [15], (d) graph-based method (GB) [37], (e) region-contrast method (RC) [38], (f) AC [39], (g) context-aware method (CA) [40], (h) low-rank matrix recovery based method (LR) [13], (i) spectral residual approach (SR) [41], (j) MSS [42], (k) our method, (l) ground truth.

The model presented in this paper considered both color and texture feature in order to overcome some shortcomings of the global contrast based models and models based on color feature only. We also introduced a more effective and logical fusion method to adjust the weights of different feature maps adaptively. We integrated high-level priors including location prior and color prior to the model to obtain a better saliency map. The experimental results show the superiority of our model in comparison with the existing models in terms of visual effect (shown in Fig 9). In the future, we will study on more complex data set, as the data set eliminating center bias. This will make huge challenge to the existing saliency detection methods, may even open up a new research direction of saliency detection.

## Supporting Information

S1 Dataset Dataset demo of THUS10000(provided by M. M. Cheng [18]), MSRA-1000 (provided by Achanta) and DB-Bruce [43]. (ZIP)
