# Supplementary figures and images for: Unified Saliency Detection Model Using Color and Texture Features (part 2 of 2)
Source: PLoS One. 2016 Feb 18;11(2):e0149328. doi: 10.1371/journal.pone.0149328 (PMC4758633; doi:10.1371/journal.pone.0149328)

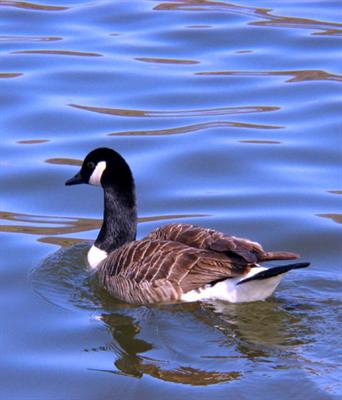

Supplement: S1 Dataset — (ZIP) [file pone.0149328.s001.zip › S1_Dataset/0_18_18734.jpg]

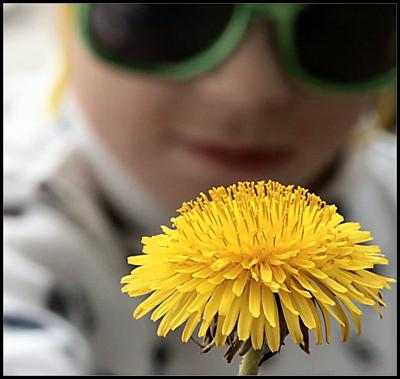

Supplement: S1 Dataset — (ZIP) [file pone.0149328.s001.zip › S1_Dataset/0_18_18852.jpg]

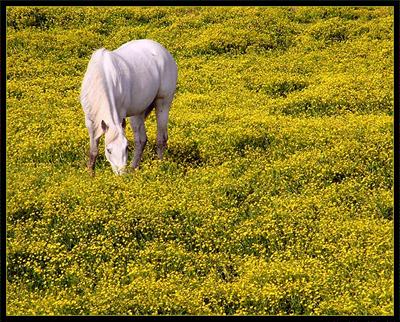

Supplement: S1 Dataset — (ZIP) [file pone.0149328.s001.zip › S1_Dataset/0_18_18763.jpg]

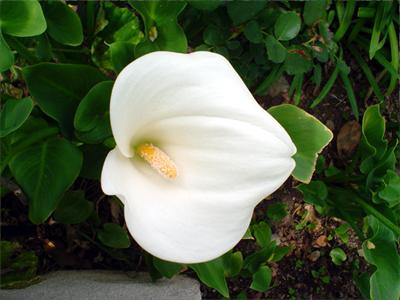

Supplement: S1 Dataset — (ZIP) [file pone.0149328.s001.zip › S1_Dataset/0_18_18880.jpg]

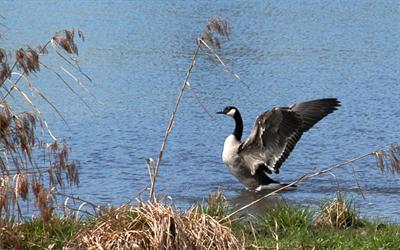

Supplement: S1 Dataset — (ZIP) [file pone.0149328.s001.zip › S1_Dataset/0_18_18957.jpg]

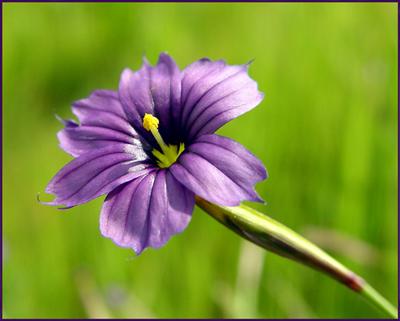

Supplement: S1 Dataset — (ZIP) [file pone.0149328.s001.zip › S1_Dataset/0_18_18961.jpg]

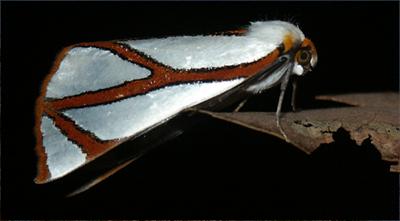

Supplement: S1 Dataset — (ZIP) [file pone.0149328.s001.zip › S1_Dataset/0_19_19011.jpg]

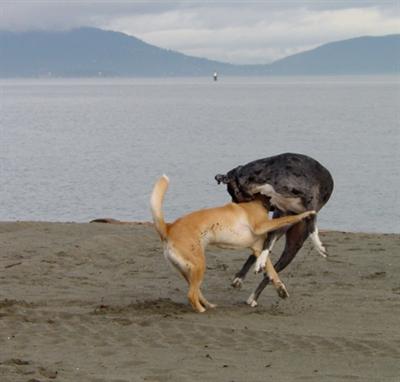

Supplement: S1 Dataset — (ZIP) [file pone.0149328.s001.zip › S1_Dataset/0_19_19025.jpg]

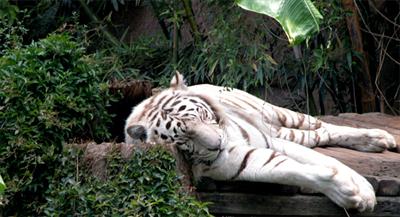

Supplement: S1 Dataset — (ZIP) [file pone.0149328.s001.zip › S1_Dataset/0_19_19068.jpg]

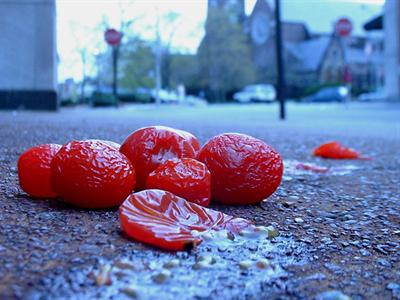

Supplement: S1 Dataset — (ZIP) [file pone.0149328.s001.zip › S1_Dataset/0_1_1004.jpg]

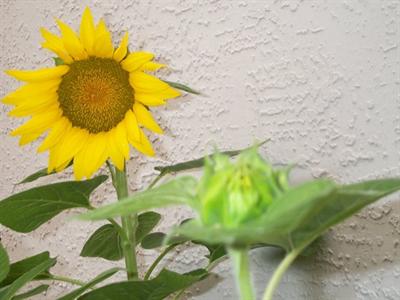

Supplement: S1 Dataset — (ZIP) [file pone.0149328.s001.zip › S1_Dataset/0_1_1039.jpg]

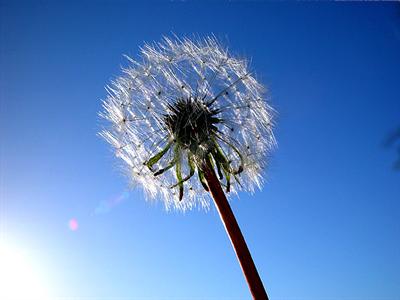

Supplement: S1 Dataset — (ZIP) [file pone.0149328.s001.zip › S1_Dataset/0_1_1339.jpg]

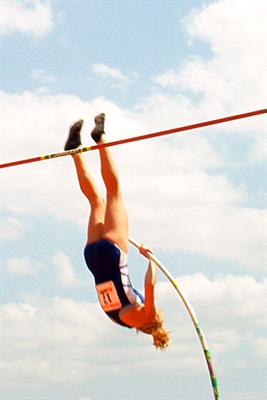

Supplement: S1 Dataset — (ZIP) [file pone.0149328.s001.zip › S1_Dataset/0_1_1611.jpg]

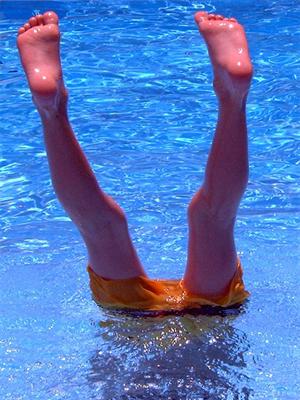

Supplement: S1 Dataset — (ZIP) [file pone.0149328.s001.zip › S1_Dataset/0_1_1626.jpg]

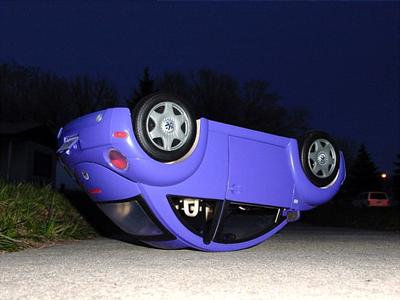

Supplement: S1 Dataset — (ZIP) [file pone.0149328.s001.zip › S1_Dataset/0_1_1650.jpg]

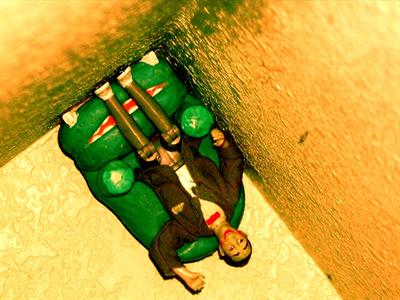

Supplement: S1 Dataset — (ZIP) [file pone.0149328.s001.zip › S1_Dataset/0_1_1664.jpg]

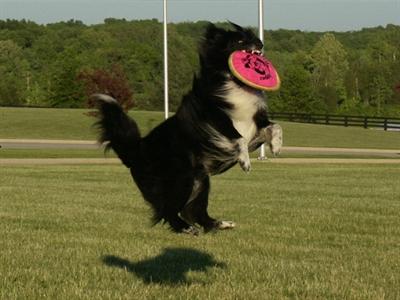

Supplement: S1 Dataset — (ZIP) [file pone.0149328.s001.zip › S1_Dataset/0_1_1696.jpg]

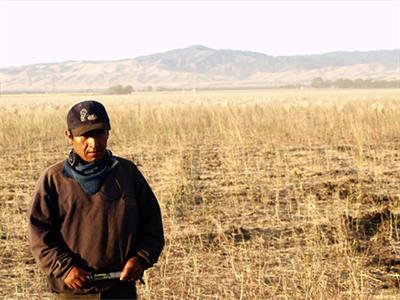

Supplement: S1 Dataset — (ZIP) [file pone.0149328.s001.zip › S1_Dataset/0_1_1865.jpg]

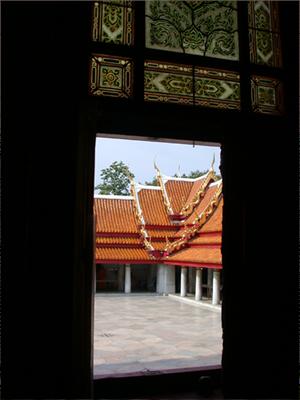

Supplement: S1 Dataset — (ZIP) [file pone.0149328.s001.zip › S1_Dataset/0_20_20573.jpg]

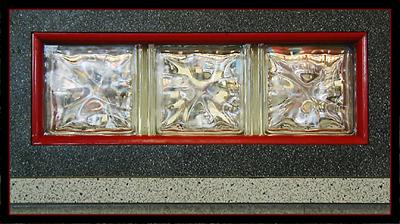

Supplement: S1 Dataset — (ZIP) [file pone.0149328.s001.zip › S1_Dataset/0_20_20656.jpg]

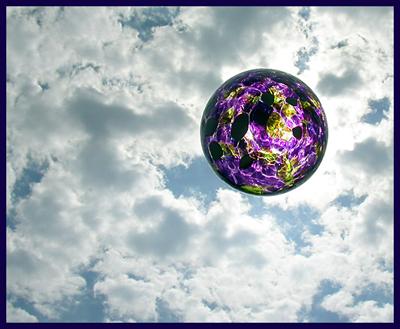

Supplement: S1 Dataset — (ZIP) [file pone.0149328.s001.zip › S1_Dataset/0_20_20772.jpg]

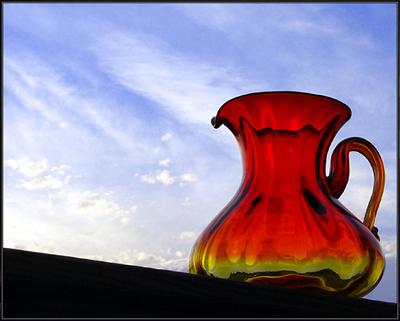

Supplement: S1 Dataset — (ZIP) [file pone.0149328.s001.zip › S1_Dataset/0_20_20783.jpg]

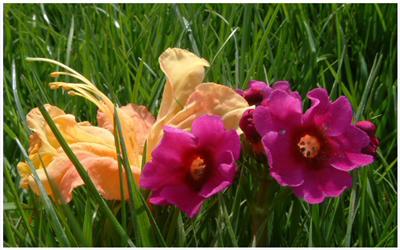

Supplement: S1 Dataset — (ZIP) [file pone.0149328.s001.zip › S1_Dataset/0_21_21001.jpg]

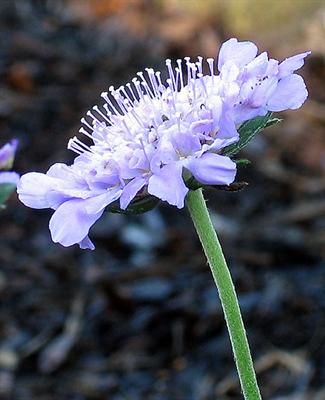

Supplement: S1 Dataset — (ZIP) [file pone.0149328.s001.zip › S1_Dataset/0_21_21087.jpg]

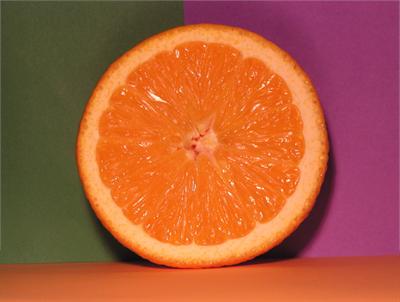

Supplement: S1 Dataset — (ZIP) [file pone.0149328.s001.zip › S1_Dataset/0_21_21093.jpg]

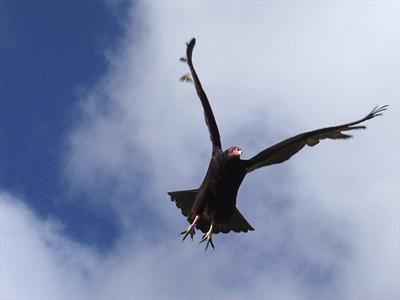

Supplement: S1 Dataset — (ZIP) [file pone.0149328.s001.zip › S1_Dataset/0_21_21135.jpg]

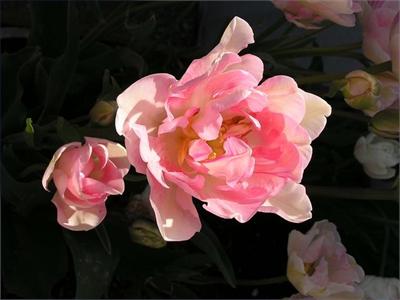

Supplement: S1 Dataset — (ZIP) [file pone.0149328.s001.zip › S1_Dataset/0_21_21147.jpg]

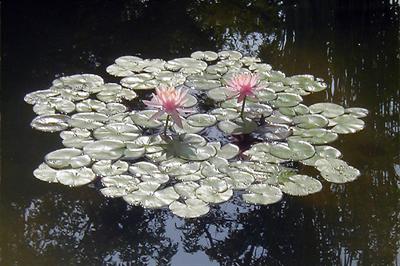

Supplement: S1 Dataset — (ZIP) [file pone.0149328.s001.zip › S1_Dataset/0_21_21299.jpg]

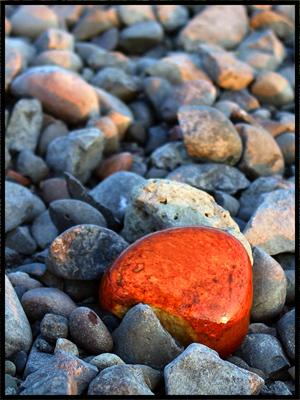

Supplement: S1 Dataset — (ZIP) [file pone.0149328.s001.zip › S1_Dataset/0_21_21332.jpg]

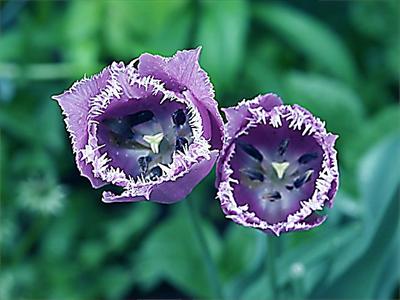

Supplement: S1 Dataset — (ZIP) [file pone.0149328.s001.zip › S1_Dataset/0_21_21413.jpg]

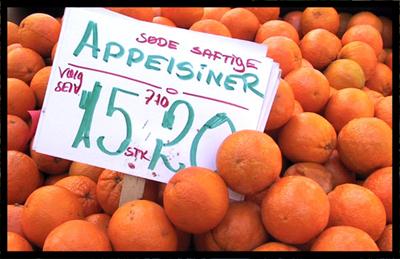

Supplement: S1 Dataset — (ZIP) [file pone.0149328.s001.zip › S1_Dataset/0_21_21422.jpg]

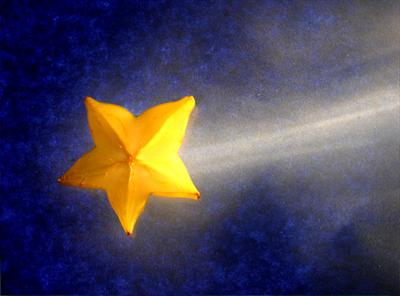

Supplement: S1 Dataset — (ZIP) [file pone.0149328.s001.zip › S1_Dataset/0_21_21703.jpg]

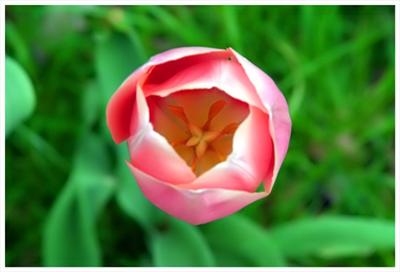

Supplement: S1 Dataset — (ZIP) [file pone.0149328.s001.zip › S1_Dataset/0_21_21938.jpg]

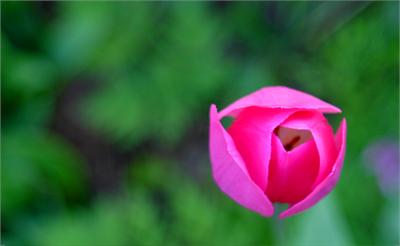

Supplement: S1 Dataset — (ZIP) [file pone.0149328.s001.zip › S1_Dataset/0_21_21974.jpg]

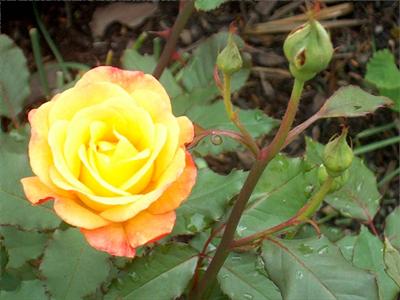

Supplement: S1 Dataset — (ZIP) [file pone.0149328.s001.zip › S1_Dataset/0_22_22218.jpg]

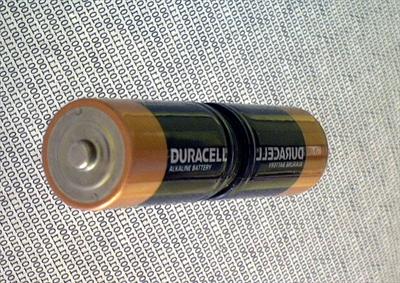

Supplement: S1 Dataset — (ZIP) [file pone.0149328.s001.zip › S1_Dataset/0_22_22047.jpg]

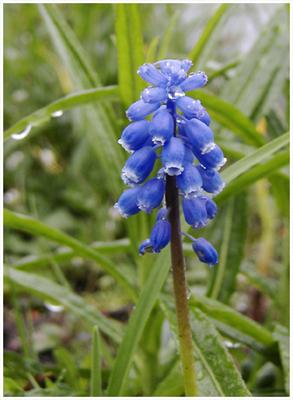

Supplement: S1 Dataset — (ZIP) [file pone.0149328.s001.zip › S1_Dataset/0_22_22234.jpg]

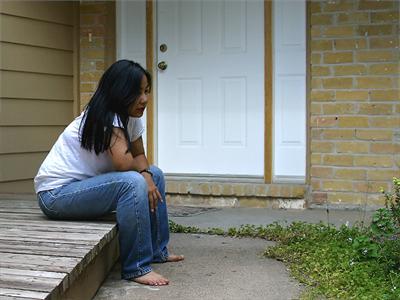

Supplement: S1 Dataset — (ZIP) [file pone.0149328.s001.zip › S1_Dataset/0_22_22838.jpg]

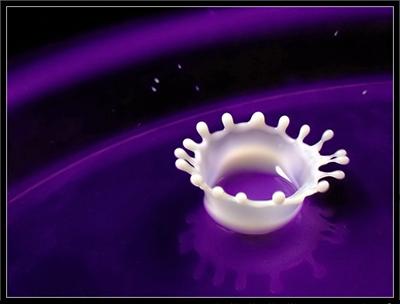

Supplement: S1 Dataset — (ZIP) [file pone.0149328.s001.zip › S1_Dataset/0_23_23666.jpg]

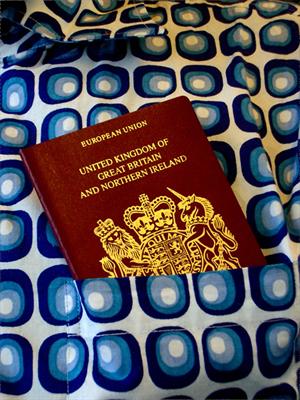

Supplement: S1 Dataset — (ZIP) [file pone.0149328.s001.zip › S1_Dataset/0_23_23003.jpg]

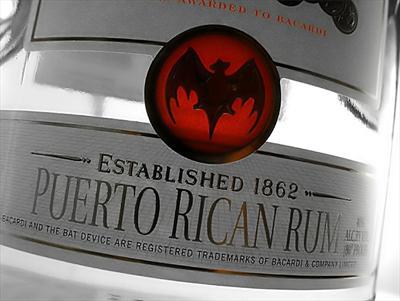

Supplement: S1 Dataset — (ZIP) [file pone.0149328.s001.zip › S1_Dataset/0_23_23683.jpg]

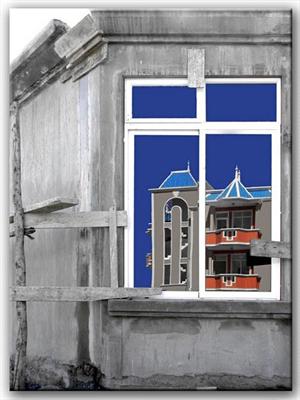

Supplement: S1 Dataset — (ZIP) [file pone.0149328.s001.zip › S1_Dataset/0_23_23697.jpg]

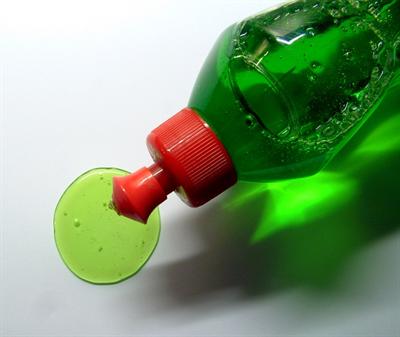

Supplement: S1 Dataset — (ZIP) [file pone.0149328.s001.zip › S1_Dataset/0_23_23748.jpg]

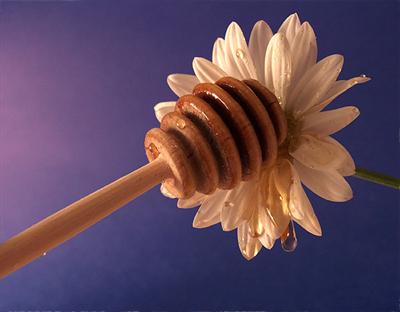

Supplement: S1 Dataset — (ZIP) [file pone.0149328.s001.zip › S1_Dataset/0_23_23817.jpg]

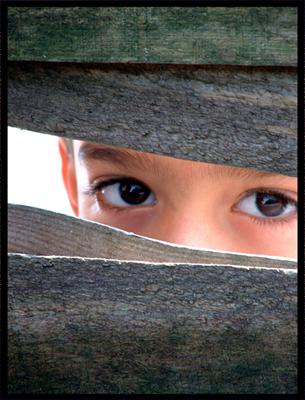

Supplement: S1 Dataset — (ZIP) [file pone.0149328.s001.zip › S1_Dataset/0_23_23934.jpg]

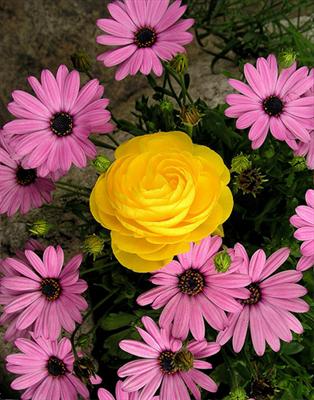

Supplement: S1 Dataset — (ZIP) [file pone.0149328.s001.zip › S1_Dataset/0_24_24071.jpg]

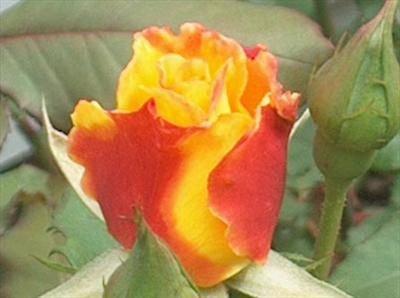

Supplement: S1 Dataset — (ZIP) [file pone.0149328.s001.zip › S1_Dataset/0_24_24196.jpg]

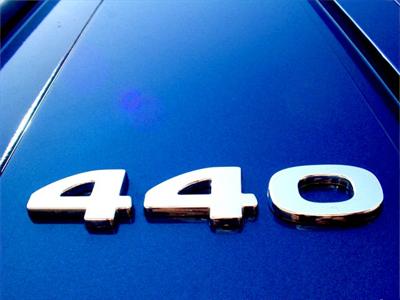

Supplement: S1 Dataset — (ZIP) [file pone.0149328.s001.zip › S1_Dataset/0_24_24209.jpg]

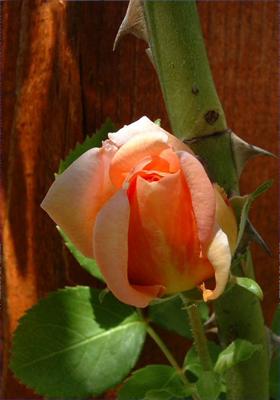

Supplement: S1 Dataset — (ZIP) [file pone.0149328.s001.zip › S1_Dataset/0_24_24234.jpg]

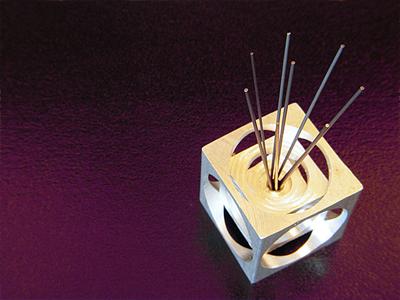

Supplement: S1 Dataset — (ZIP) [file pone.0149328.s001.zip › S1_Dataset/0_24_24256.jpg]

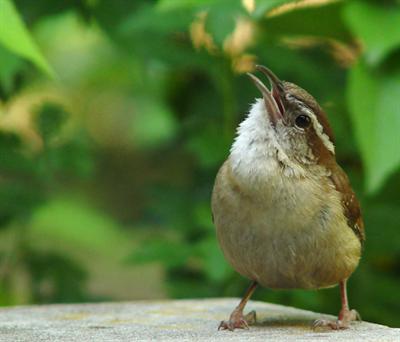

Supplement: S1 Dataset — (ZIP) [file pone.0149328.s001.zip › S1_Dataset/0_24_24455.jpg]

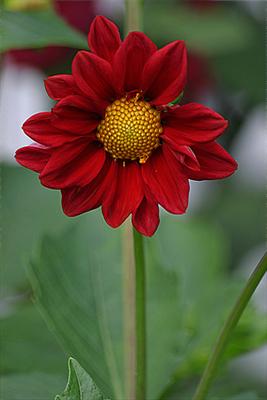

Supplement: S1 Dataset — (ZIP) [file pone.0149328.s001.zip › S1_Dataset/0_24_24500.jpg]

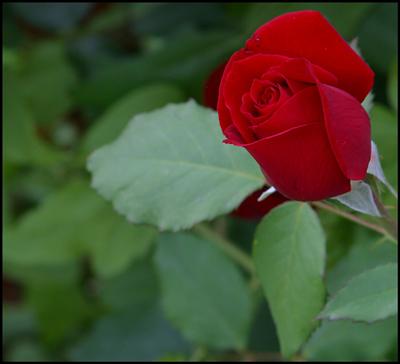

Supplement: S1 Dataset — (ZIP) [file pone.0149328.s001.zip › S1_Dataset/0_24_24670.jpg]

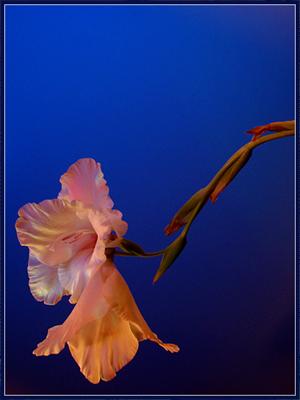

Supplement: S1 Dataset — (ZIP) [file pone.0149328.s001.zip › S1_Dataset/0_24_24771.jpg]

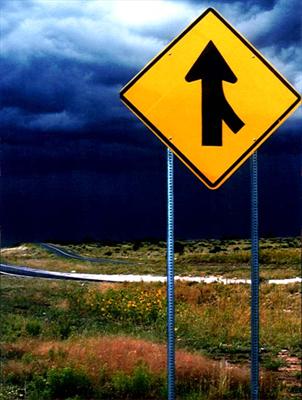

Supplement: S1 Dataset — (ZIP) [file pone.0149328.s001.zip › S1_Dataset/0_24_24829.jpg]

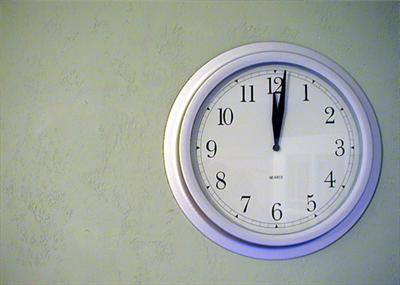

Supplement: S1 Dataset — (ZIP) [file pone.0149328.s001.zip › S1_Dataset/0_24_24861.jpg]

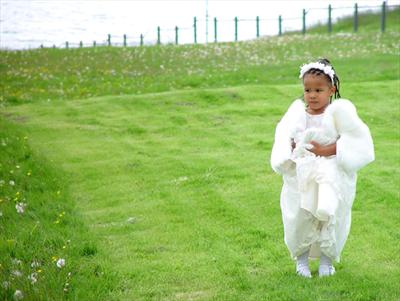

Supplement: S1 Dataset — (ZIP) [file pone.0149328.s001.zip › S1_Dataset/0_24_24918.jpg]

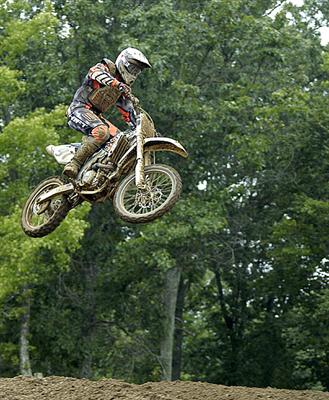

Supplement: S1 Dataset — (ZIP) [file pone.0149328.s001.zip › S1_Dataset/0_24_24965.jpg]

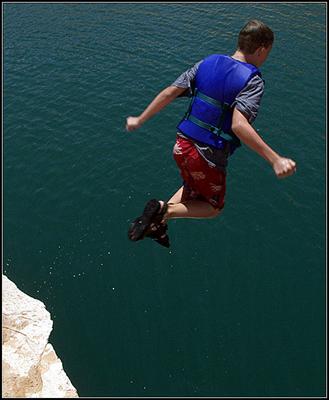

Supplement: S1 Dataset — (ZIP) [file pone.0149328.s001.zip › S1_Dataset/0_24_24973.jpg]

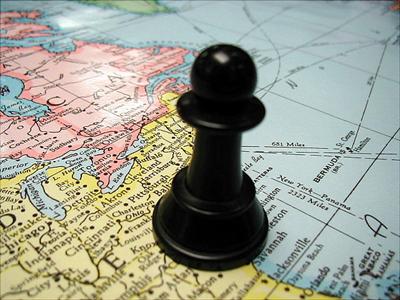

Supplement: S1 Dataset — (ZIP) [file pone.0149328.s001.zip › S1_Dataset/0_25_25057.jpg]

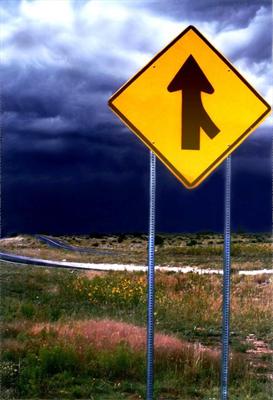

Supplement: S1 Dataset — (ZIP) [file pone.0149328.s001.zip › S1_Dataset/0_25_25064.jpg]

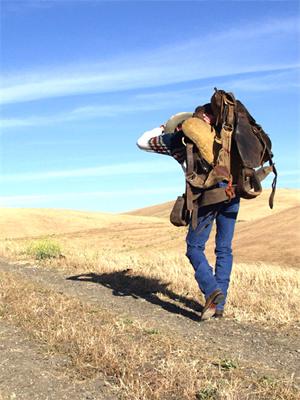

Supplement: S1 Dataset — (ZIP) [file pone.0149328.s001.zip › S1_Dataset/0_2_2276.jpg]

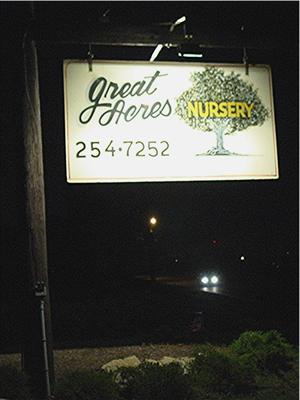

Supplement: S1 Dataset — (ZIP) [file pone.0149328.s001.zip › S1_Dataset/0_2_2304.jpg]

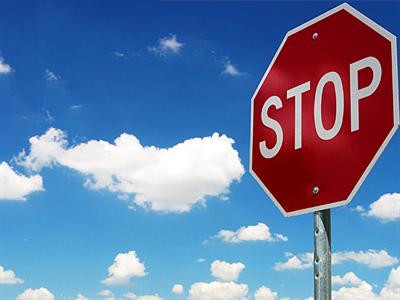

Supplement: S1 Dataset — (ZIP) [file pone.0149328.s001.zip › S1_Dataset/0_2_2310.jpg]

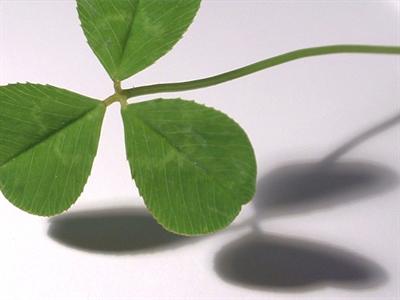

Supplement: S1 Dataset — (ZIP) [file pone.0149328.s001.zip › S1_Dataset/0_2_2551.jpg]

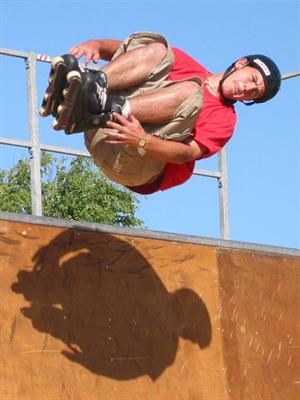

Supplement: S1 Dataset — (ZIP) [file pone.0149328.s001.zip › S1_Dataset/0_2_2580.jpg]

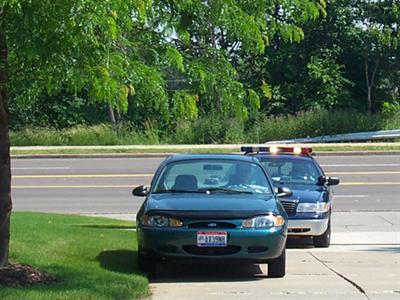

Supplement: S1 Dataset — (ZIP) [file pone.0149328.s001.zip › S1_Dataset/0_2_2721.jpg]

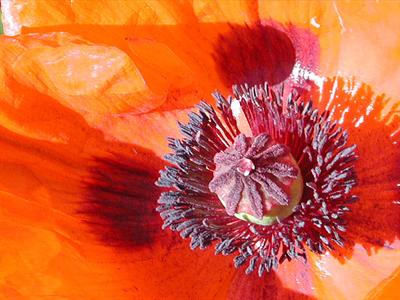

Supplement: S1 Dataset — (ZIP) [file pone.0149328.s001.zip › S1_Dataset/0_3_3183.jpg]

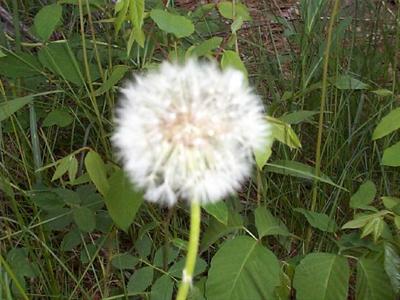

Supplement: S1 Dataset — (ZIP) [file pone.0149328.s001.zip › S1_Dataset/0_3_3294.jpg]

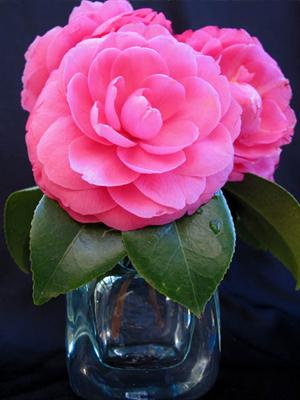

Supplement: S1 Dataset — (ZIP) [file pone.0149328.s001.zip › S1_Dataset/0_3_3308.jpg]

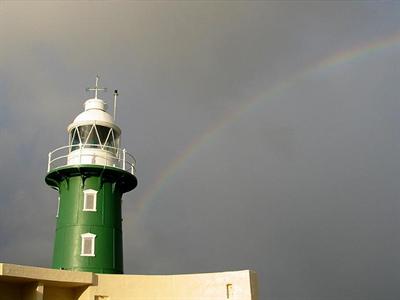

Supplement: S1 Dataset — (ZIP) [file pone.0149328.s001.zip › S1_Dataset/0_3_3317.jpg]

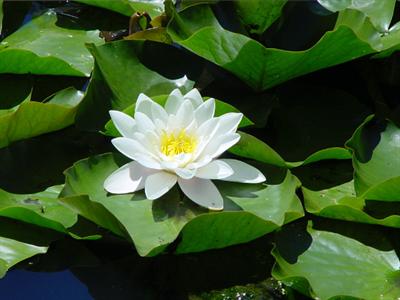

Supplement: S1 Dataset — (ZIP) [file pone.0149328.s001.zip › S1_Dataset/0_3_3327.jpg]

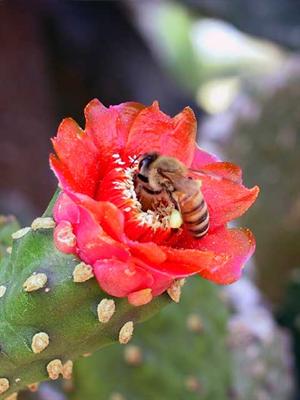

Supplement: S1 Dataset — (ZIP) [file pone.0149328.s001.zip › S1_Dataset/0_3_3344.jpg]

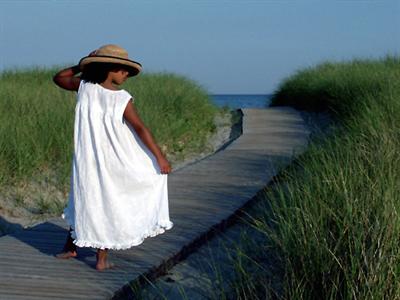

Supplement: S1 Dataset — (ZIP) [file pone.0149328.s001.zip › S1_Dataset/0_3_3362.jpg]

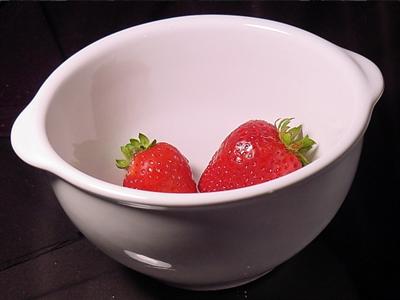

Supplement: S1 Dataset — (ZIP) [file pone.0149328.s001.zip › S1_Dataset/0_3_3434.jpg]

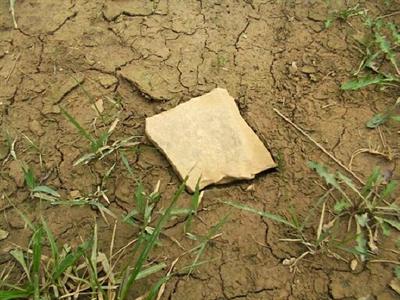

Supplement: S1 Dataset — (ZIP) [file pone.0149328.s001.zip › S1_Dataset/0_3_3514.jpg]

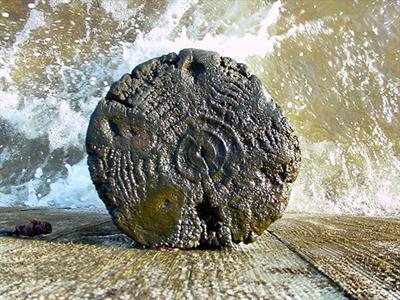

Supplement: S1 Dataset — (ZIP) [file pone.0149328.s001.zip › S1_Dataset/0_3_3524.jpg]

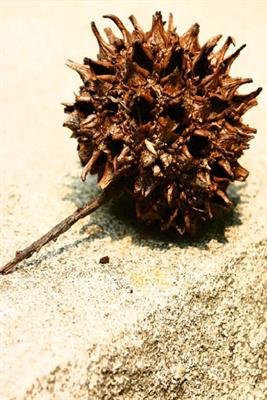

Supplement: S1 Dataset — (ZIP) [file pone.0149328.s001.zip › S1_Dataset/0_3_3551.jpg]

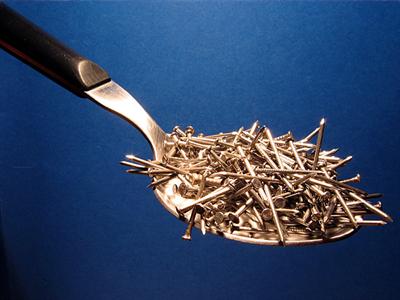

Supplement: S1 Dataset — (ZIP) [file pone.0149328.s001.zip › S1_Dataset/0_3_3559.jpg]

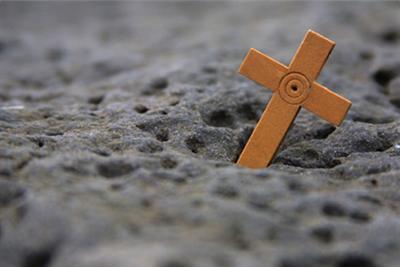

Supplement: S1 Dataset — (ZIP) [file pone.0149328.s001.zip › S1_Dataset/0_3_3654.jpg]

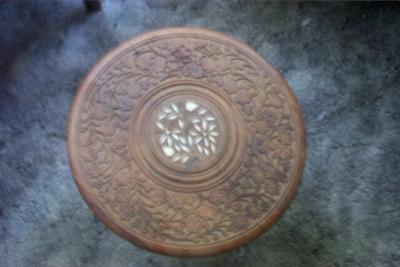

Supplement: S1 Dataset — (ZIP) [file pone.0149328.s001.zip › S1_Dataset/0_3_3692.jpg]

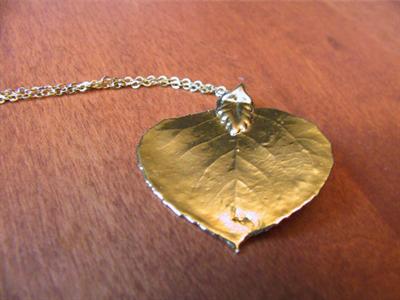

Supplement: S1 Dataset — (ZIP) [file pone.0149328.s001.zip › S1_Dataset/0_3_3732.jpg]

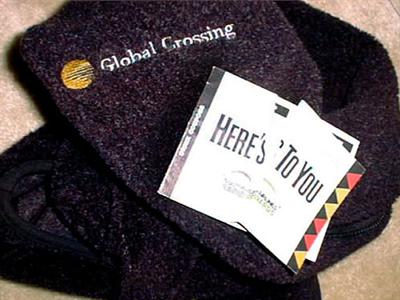

Supplement: S1 Dataset — (ZIP) [file pone.0149328.s001.zip › S1_Dataset/0_3_3914.jpg]

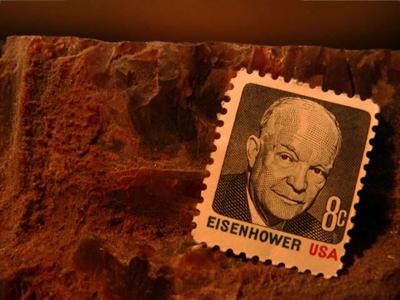

Supplement: S1 Dataset — (ZIP) [file pone.0149328.s001.zip › S1_Dataset/0_4_4038.jpg]

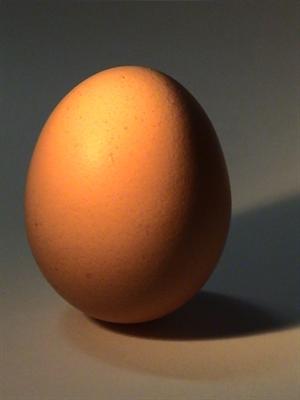

Supplement: S1 Dataset — (ZIP) [file pone.0149328.s001.zip › S1_Dataset/0_4_4283.jpg]

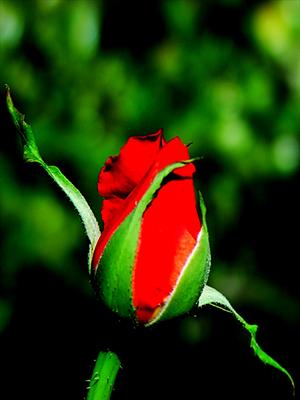

Supplement: S1 Dataset — (ZIP) [file pone.0149328.s001.zip › S1_Dataset/0_4_4328.jpg]

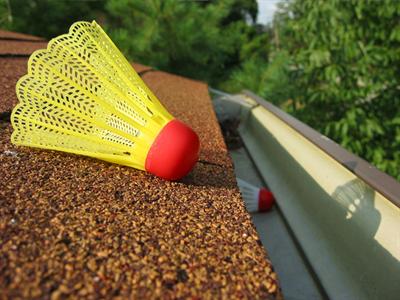

Supplement: S1 Dataset — (ZIP) [file pone.0149328.s001.zip › S1_Dataset/0_4_4870.jpg]

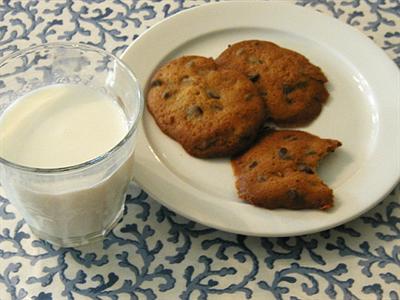

Supplement: S1 Dataset — (ZIP) [file pone.0149328.s001.zip › S1_Dataset/0_4_4976.jpg]

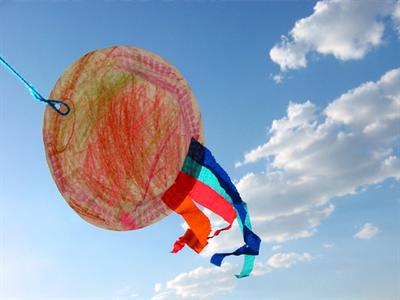

Supplement: S1 Dataset — (ZIP) [file pone.0149328.s001.zip › S1_Dataset/0_5_5010.jpg]

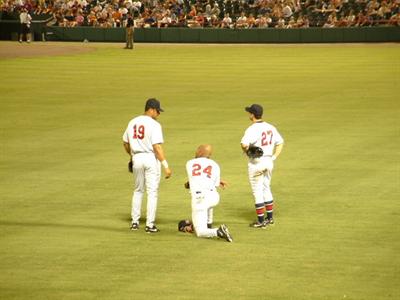

Supplement: S1 Dataset — (ZIP) [file pone.0149328.s001.zip › S1_Dataset/0_5_5108.jpg]

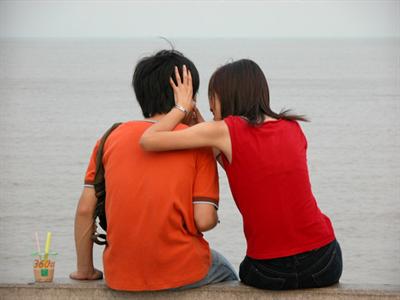

Supplement: S1 Dataset — (ZIP) [file pone.0149328.s001.zip › S1_Dataset/0_5_5189.jpg]

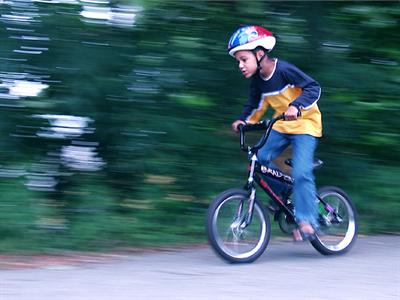

Supplement: S1 Dataset — (ZIP) [file pone.0149328.s001.zip › S1_Dataset/0_5_5255.jpg]

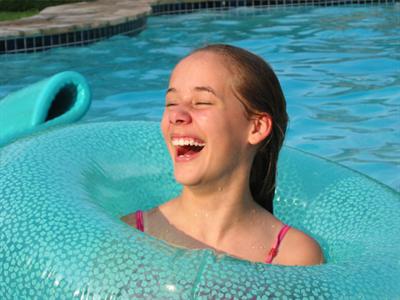

Supplement: S1 Dataset — (ZIP) [file pone.0149328.s001.zip › S1_Dataset/0_5_5303.jpg]

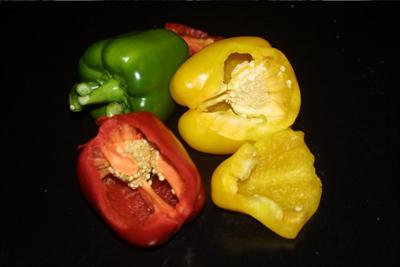

Supplement: S1 Dataset — (ZIP) [file pone.0149328.s001.zip › S1_Dataset/0_5_5318.jpg]

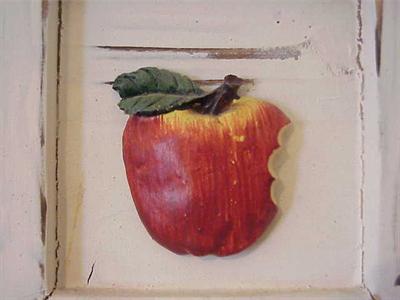

Supplement: S1 Dataset — (ZIP) [file pone.0149328.s001.zip › S1_Dataset/0_5_5416.jpg]

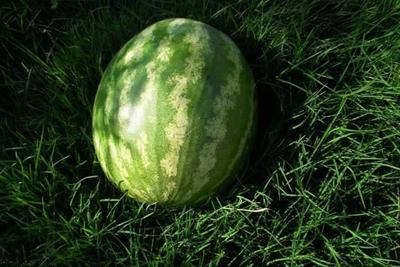

Supplement: S1 Dataset — (ZIP) [file pone.0149328.s001.zip › S1_Dataset/0_5_5463.jpg]

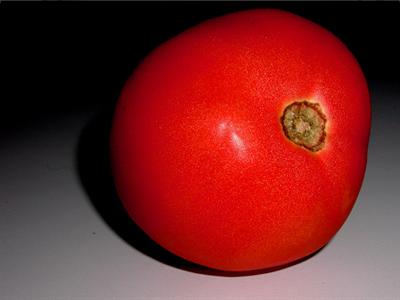

Supplement: S1 Dataset — (ZIP) [file pone.0149328.s001.zip › S1_Dataset/0_5_5528.jpg]

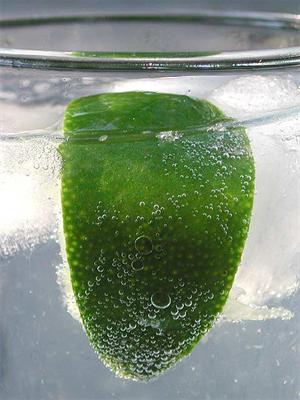

Supplement: S1 Dataset — (ZIP) [file pone.0149328.s001.zip › S1_Dataset/0_5_5566.jpg]

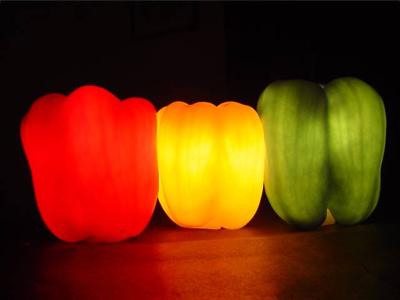

Supplement: S1 Dataset — (ZIP) [file pone.0149328.s001.zip › S1_Dataset/0_5_5586.jpg]

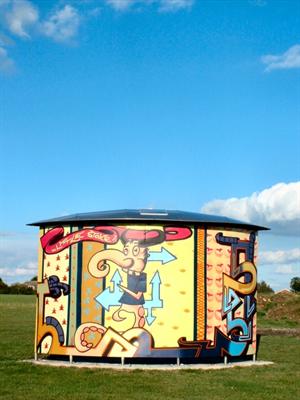

Supplement: S1 Dataset — (ZIP) [file pone.0149328.s001.zip › S1_Dataset/0_5_5634.jpg]
