# Supplementary figures and images for: Unified Saliency Detection Model Using Color and Texture Features (part 1 of 2)
Source: PLoS One. 2016 Feb 18;11(2):e0149328. doi: 10.1371/journal.pone.0149328 (PMC4758633; doi:10.1371/journal.pone.0149328)

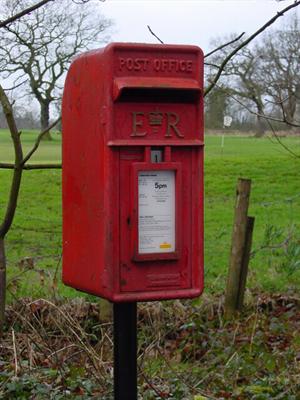

Supplement: S1 Dataset — (ZIP) [file pone.0149328.s001.zip › S1_Dataset/0_0_272.jpg]

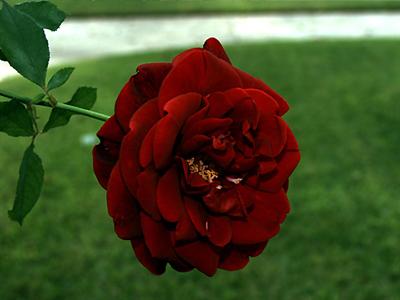

Supplement: S1 Dataset — (ZIP) [file pone.0149328.s001.zip › S1_Dataset/0_0_280.jpg]

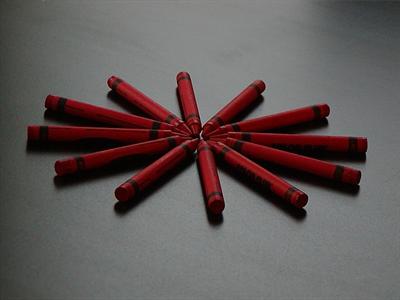

Supplement: S1 Dataset — (ZIP) [file pone.0149328.s001.zip › S1_Dataset/0_0_284.jpg]

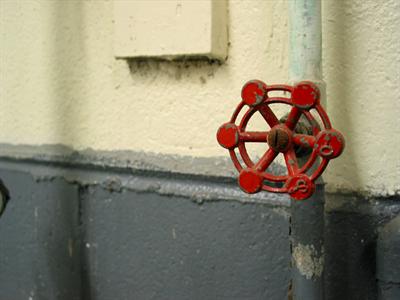

Supplement: S1 Dataset — (ZIP) [file pone.0149328.s001.zip › S1_Dataset/0_0_307.jpg]

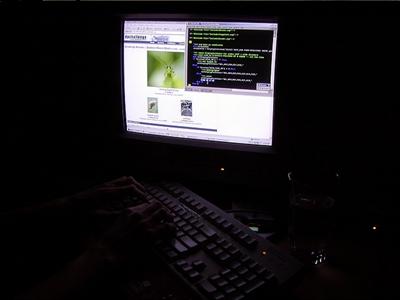

Supplement: S1 Dataset — (ZIP) [file pone.0149328.s001.zip › S1_Dataset/0_0_355.jpg]

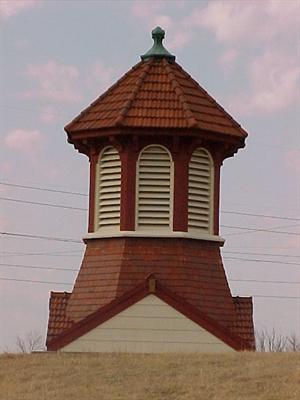

Supplement: S1 Dataset — (ZIP) [file pone.0149328.s001.zip › S1_Dataset/0_0_547.jpg]

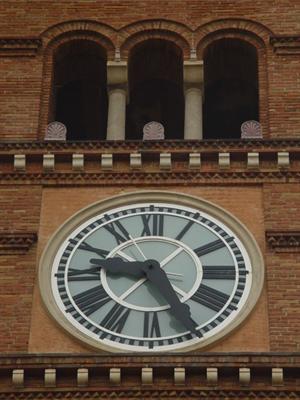

Supplement: S1 Dataset — (ZIP) [file pone.0149328.s001.zip › S1_Dataset/0_0_579.jpg]

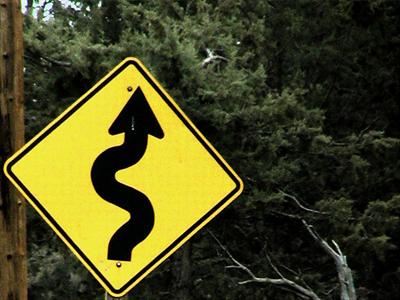

Supplement: S1 Dataset — (ZIP) [file pone.0149328.s001.zip › S1_Dataset/0_0_735.jpg]

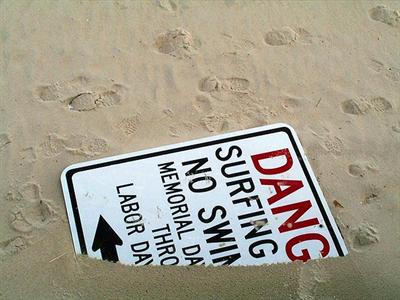

Supplement: S1 Dataset — (ZIP) [file pone.0149328.s001.zip › S1_Dataset/0_0_77.jpg]

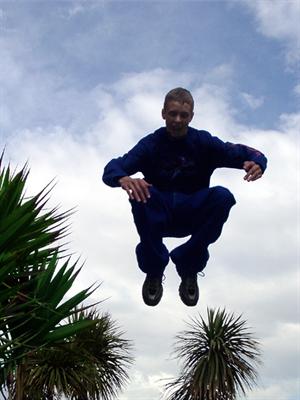

Supplement: S1 Dataset — (ZIP) [file pone.0149328.s001.zip › S1_Dataset/0_0_818.jpg]

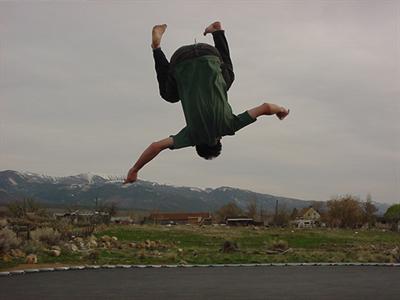

Supplement: S1 Dataset — (ZIP) [file pone.0149328.s001.zip › S1_Dataset/0_0_899.jpg]

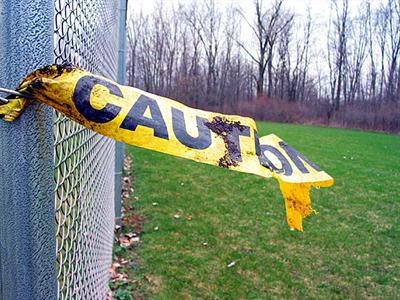

Supplement: S1 Dataset — (ZIP) [file pone.0149328.s001.zip › S1_Dataset/0_0_840.jpg]

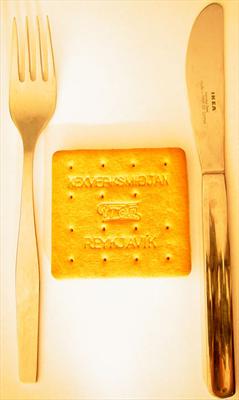

Supplement: S1 Dataset — (ZIP) [file pone.0149328.s001.zip › S1_Dataset/0_10_10938.jpg]

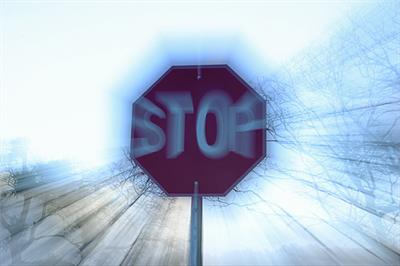

Supplement: S1 Dataset — (ZIP) [file pone.0149328.s001.zip › S1_Dataset/0_11_11060.jpg]

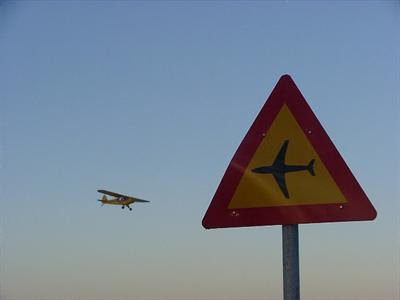

Supplement: S1 Dataset — (ZIP) [file pone.0149328.s001.zip › S1_Dataset/0_11_11136.jpg]

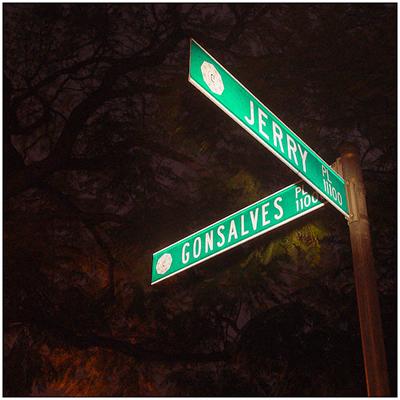

Supplement: S1 Dataset — (ZIP) [file pone.0149328.s001.zip › S1_Dataset/0_11_11179.jpg]

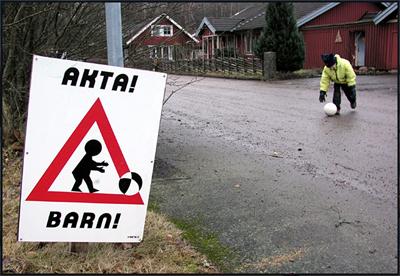

Supplement: S1 Dataset — (ZIP) [file pone.0149328.s001.zip › S1_Dataset/0_11_11219.jpg]

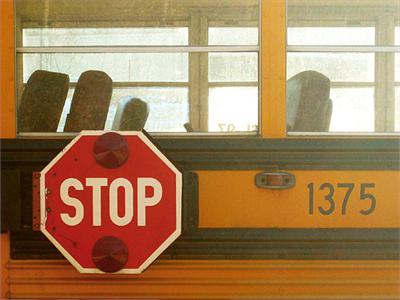

Supplement: S1 Dataset — (ZIP) [file pone.0149328.s001.zip › S1_Dataset/0_11_11281.jpg]

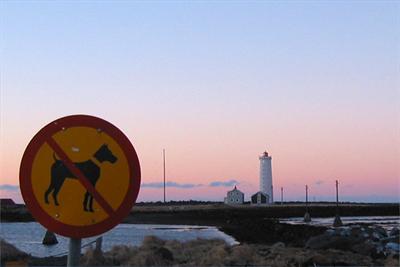

Supplement: S1 Dataset — (ZIP) [file pone.0149328.s001.zip › S1_Dataset/0_11_11297.jpg]

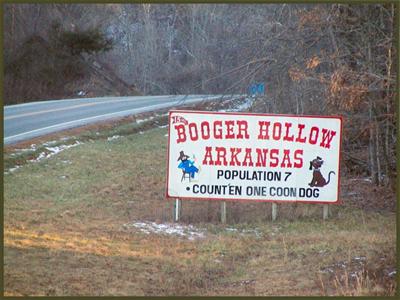

Supplement: S1 Dataset — (ZIP) [file pone.0149328.s001.zip › S1_Dataset/0_11_11298.jpg]

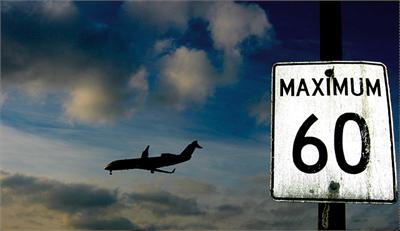

Supplement: S1 Dataset — (ZIP) [file pone.0149328.s001.zip › S1_Dataset/0_11_11325.jpg]

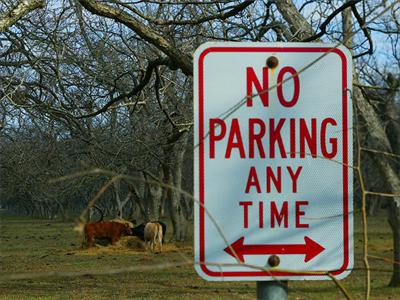

Supplement: S1 Dataset — (ZIP) [file pone.0149328.s001.zip › S1_Dataset/0_11_11313.jpg]

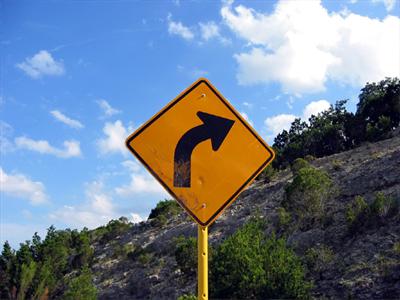

Supplement: S1 Dataset — (ZIP) [file pone.0149328.s001.zip › S1_Dataset/0_11_11346.jpg]

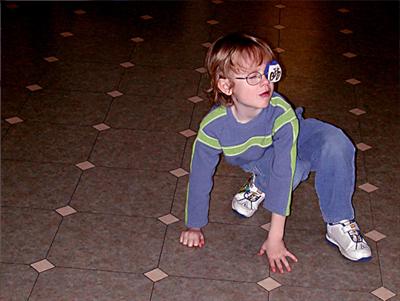

Supplement: S1 Dataset — (ZIP) [file pone.0149328.s001.zip › S1_Dataset/0_11_11459.jpg]

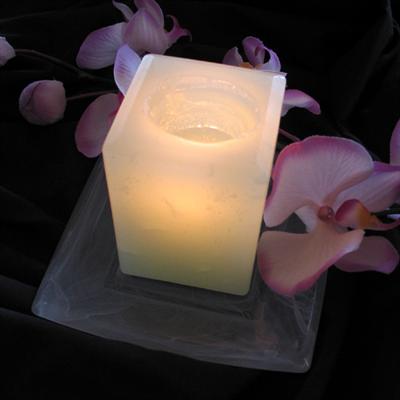

Supplement: S1 Dataset — (ZIP) [file pone.0149328.s001.zip › S1_Dataset/0_11_11480.jpg]

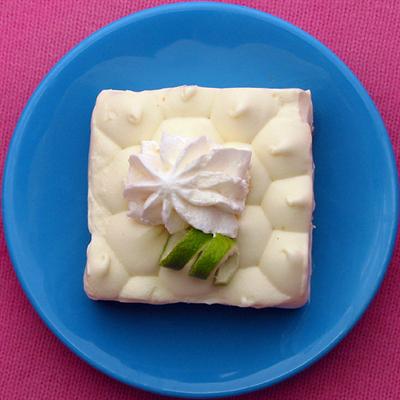

Supplement: S1 Dataset — (ZIP) [file pone.0149328.s001.zip › S1_Dataset/0_11_11517.jpg]

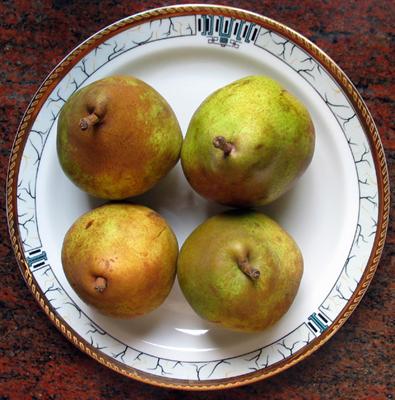

Supplement: S1 Dataset — (ZIP) [file pone.0149328.s001.zip › S1_Dataset/0_11_11533.jpg]

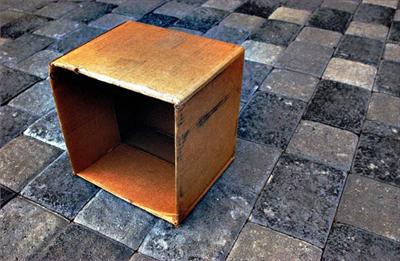

Supplement: S1 Dataset — (ZIP) [file pone.0149328.s001.zip › S1_Dataset/0_11_11557.jpg]

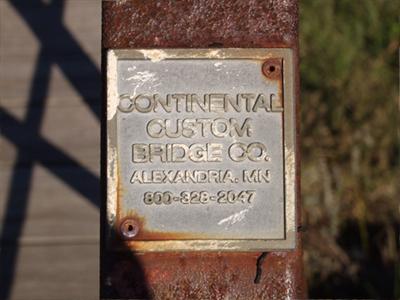

Supplement: S1 Dataset — (ZIP) [file pone.0149328.s001.zip › S1_Dataset/0_11_11571.jpg]

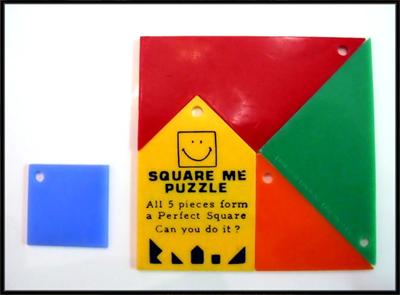

Supplement: S1 Dataset — (ZIP) [file pone.0149328.s001.zip › S1_Dataset/0_11_11650.jpg]

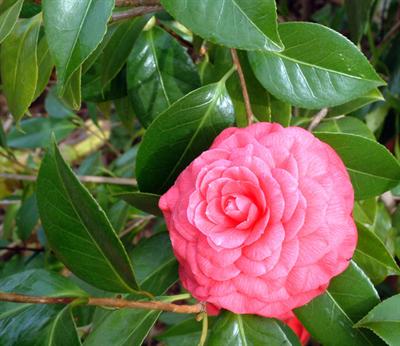

Supplement: S1 Dataset — (ZIP) [file pone.0149328.s001.zip › S1_Dataset/0_11_11830.jpg]

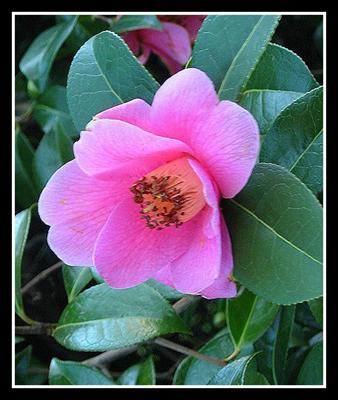

Supplement: S1 Dataset — (ZIP) [file pone.0149328.s001.zip › S1_Dataset/0_11_11852.jpg]

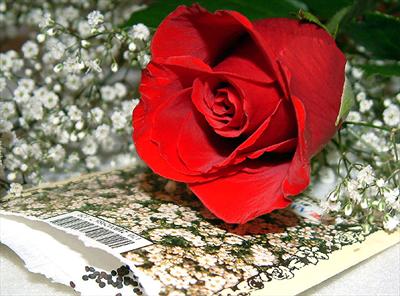

Supplement: S1 Dataset — (ZIP) [file pone.0149328.s001.zip › S1_Dataset/0_11_11875.jpg]

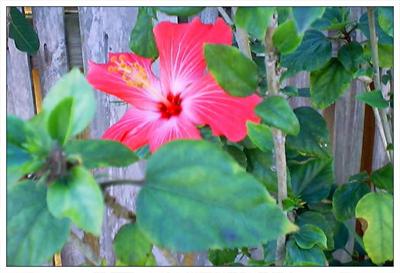

Supplement: S1 Dataset — (ZIP) [file pone.0149328.s001.zip › S1_Dataset/0_11_11881.jpg]

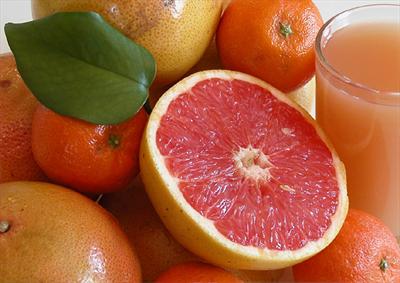

Supplement: S1 Dataset — (ZIP) [file pone.0149328.s001.zip › S1_Dataset/0_11_11987.jpg]

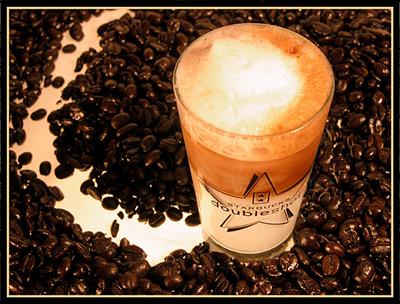

Supplement: S1 Dataset — (ZIP) [file pone.0149328.s001.zip › S1_Dataset/0_12_12048.jpg]

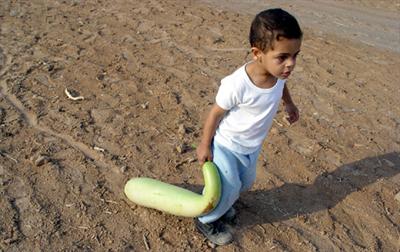

Supplement: S1 Dataset — (ZIP) [file pone.0149328.s001.zip › S1_Dataset/0_12_12072.jpg]

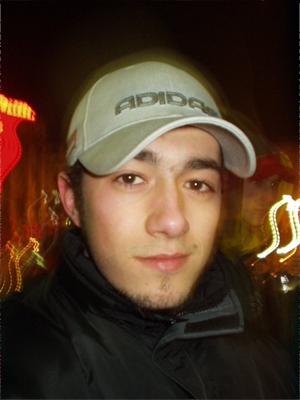

Supplement: S1 Dataset — (ZIP) [file pone.0149328.s001.zip › S1_Dataset/0_12_12144.jpg]

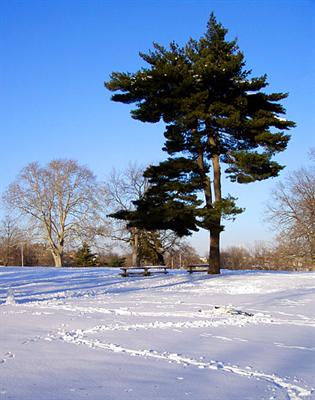

Supplement: S1 Dataset — (ZIP) [file pone.0149328.s001.zip › S1_Dataset/0_12_12171.jpg]

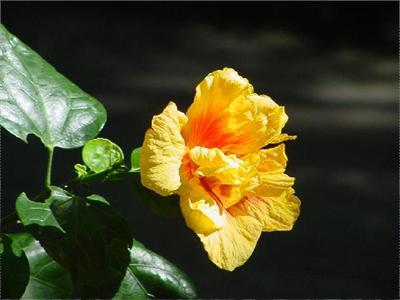

Supplement: S1 Dataset — (ZIP) [file pone.0149328.s001.zip › S1_Dataset/0_12_12344.jpg]

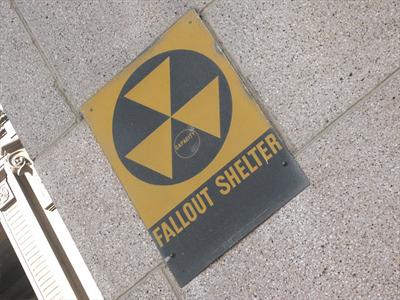

Supplement: S1 Dataset — (ZIP) [file pone.0149328.s001.zip › S1_Dataset/0_12_12484.jpg]

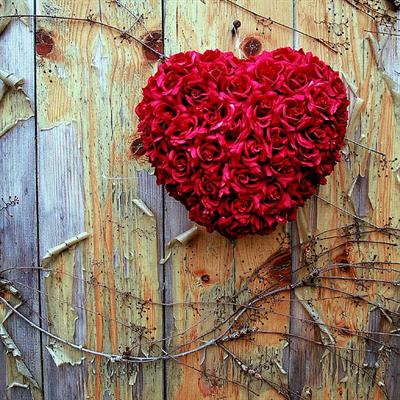

Supplement: S1 Dataset — (ZIP) [file pone.0149328.s001.zip › S1_Dataset/0_12_12435.jpg]

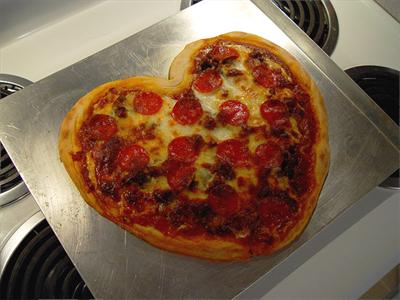

Supplement: S1 Dataset — (ZIP) [file pone.0149328.s001.zip › S1_Dataset/0_12_12518.jpg]

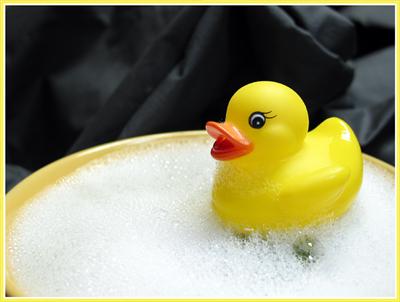

Supplement: S1 Dataset — (ZIP) [file pone.0149328.s001.zip › S1_Dataset/0_12_12597.jpg]

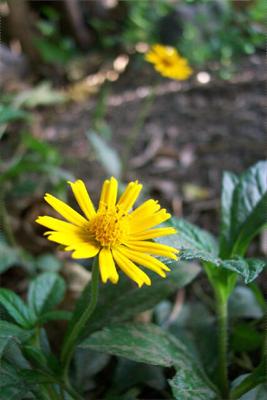

Supplement: S1 Dataset — (ZIP) [file pone.0149328.s001.zip › S1_Dataset/0_12_12619.jpg]

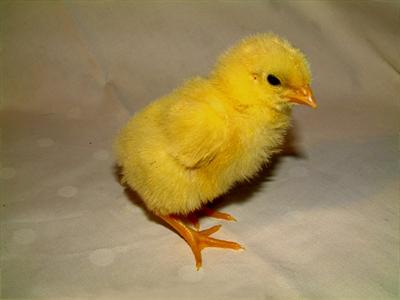

Supplement: S1 Dataset — (ZIP) [file pone.0149328.s001.zip › S1_Dataset/0_12_12649.jpg]

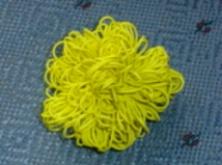

Supplement: S1 Dataset — (ZIP) [file pone.0149328.s001.zip › S1_Dataset/0_12_12705.jpg]

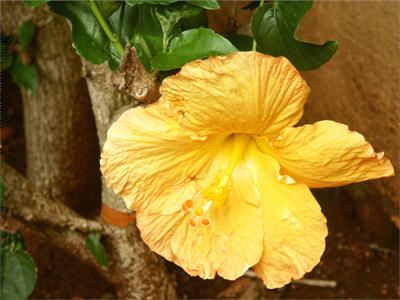

Supplement: S1 Dataset — (ZIP) [file pone.0149328.s001.zip › S1_Dataset/0_12_12750.jpg]

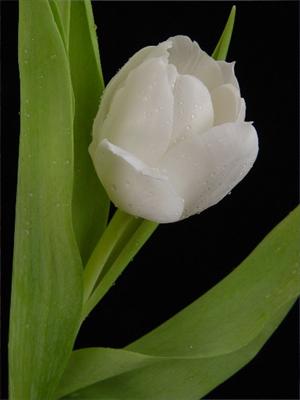

Supplement: S1 Dataset — (ZIP) [file pone.0149328.s001.zip › S1_Dataset/0_12_12816.jpg]

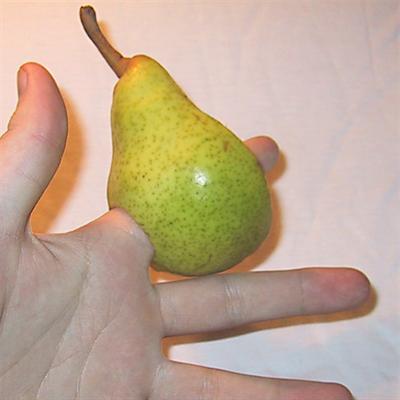

Supplement: S1 Dataset — (ZIP) [file pone.0149328.s001.zip › S1_Dataset/0_12_12833.jpg]

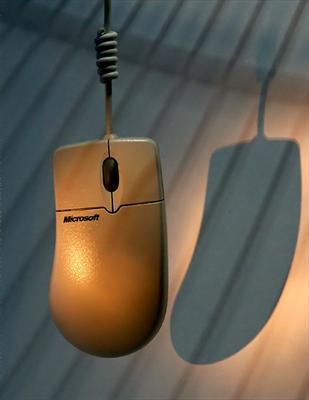

Supplement: S1 Dataset — (ZIP) [file pone.0149328.s001.zip › S1_Dataset/0_12_12892.jpg]

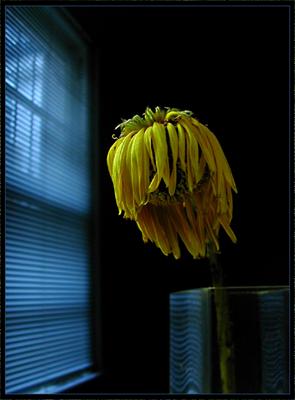

Supplement: S1 Dataset — (ZIP) [file pone.0149328.s001.zip › S1_Dataset/0_12_12923.jpg]

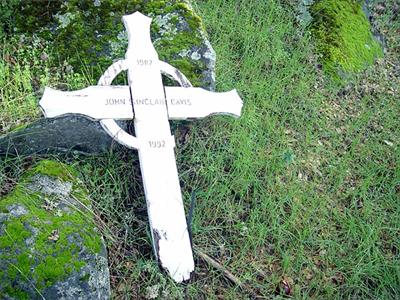

Supplement: S1 Dataset — (ZIP) [file pone.0149328.s001.zip › S1_Dataset/0_12_12921.jpg]

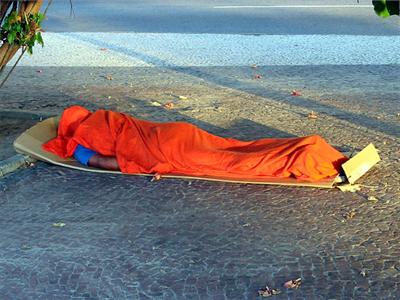

Supplement: S1 Dataset — (ZIP) [file pone.0149328.s001.zip › S1_Dataset/0_13_13036.jpg]

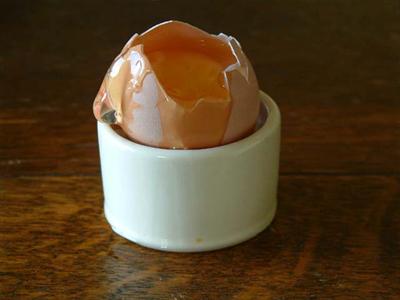

Supplement: S1 Dataset — (ZIP) [file pone.0149328.s001.zip › S1_Dataset/0_13_13198.jpg]

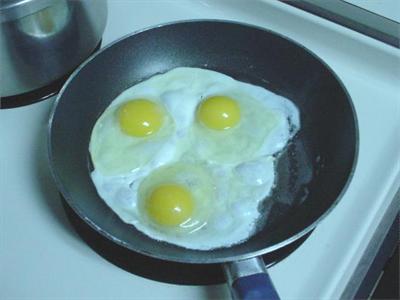

Supplement: S1 Dataset — (ZIP) [file pone.0149328.s001.zip › S1_Dataset/0_13_13235.jpg]

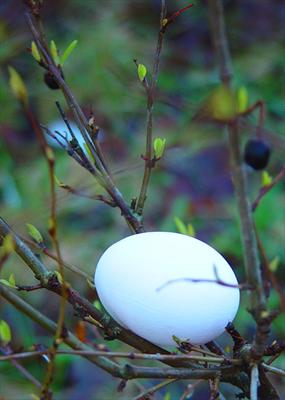

Supplement: S1 Dataset — (ZIP) [file pone.0149328.s001.zip › S1_Dataset/0_13_13308.jpg]

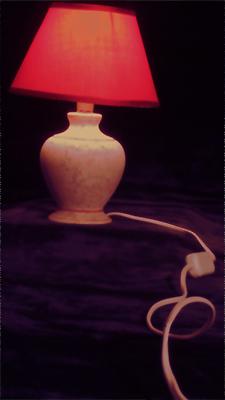

Supplement: S1 Dataset — (ZIP) [file pone.0149328.s001.zip › S1_Dataset/0_13_13420.jpg]

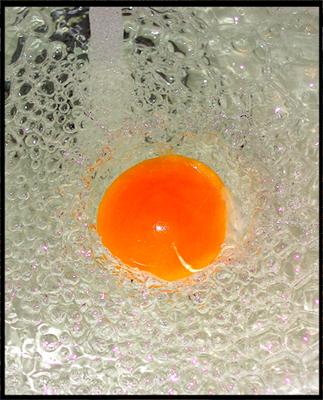

Supplement: S1 Dataset — (ZIP) [file pone.0149328.s001.zip › S1_Dataset/0_13_13339.jpg]

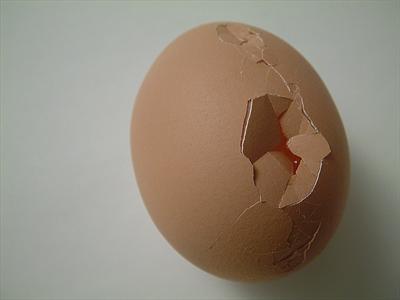

Supplement: S1 Dataset — (ZIP) [file pone.0149328.s001.zip › S1_Dataset/0_13_13515.jpg]

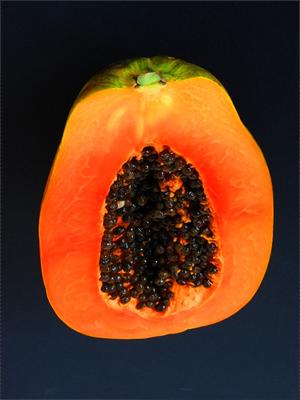

Supplement: S1 Dataset — (ZIP) [file pone.0149328.s001.zip › S1_Dataset/0_13_13553.jpg]

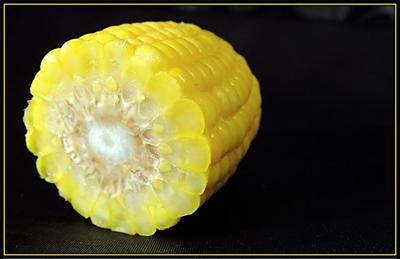

Supplement: S1 Dataset — (ZIP) [file pone.0149328.s001.zip › S1_Dataset/0_13_13885.jpg]

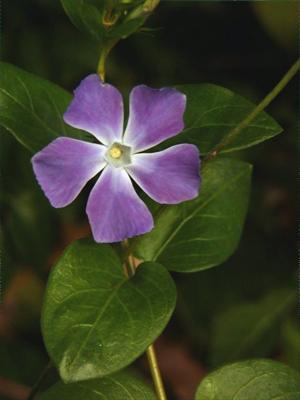

Supplement: S1 Dataset — (ZIP) [file pone.0149328.s001.zip › S1_Dataset/0_14_14532.jpg]

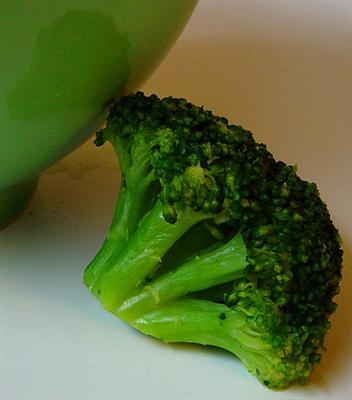

Supplement: S1 Dataset — (ZIP) [file pone.0149328.s001.zip › S1_Dataset/0_14_14991.jpg]

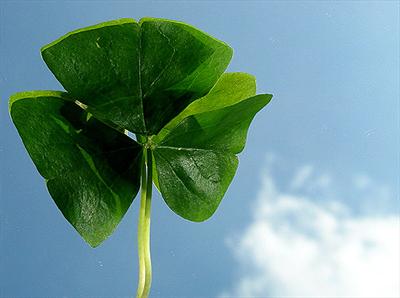

Supplement: S1 Dataset — (ZIP) [file pone.0149328.s001.zip › S1_Dataset/0_15_15030.jpg]

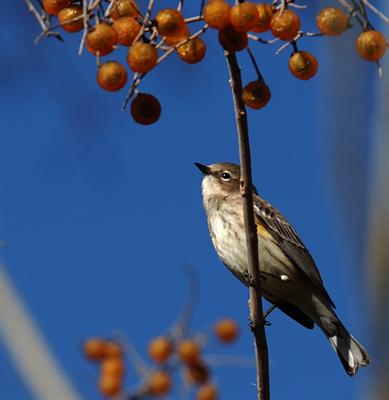

Supplement: S1 Dataset — (ZIP) [file pone.0149328.s001.zip › S1_Dataset/0_15_15264.jpg]

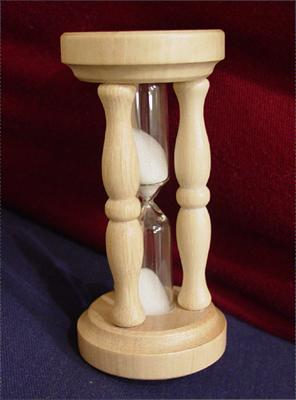

Supplement: S1 Dataset — (ZIP) [file pone.0149328.s001.zip › S1_Dataset/0_15_15522.jpg]

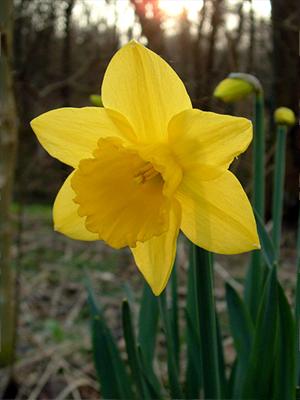

Supplement: S1 Dataset — (ZIP) [file pone.0149328.s001.zip › S1_Dataset/0_15_15620.jpg]

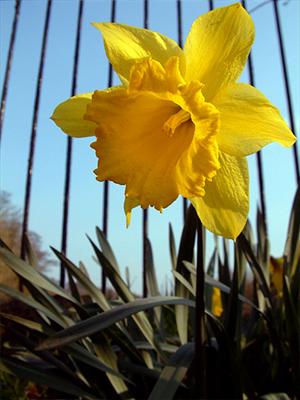

Supplement: S1 Dataset — (ZIP) [file pone.0149328.s001.zip › S1_Dataset/0_15_15622.jpg]

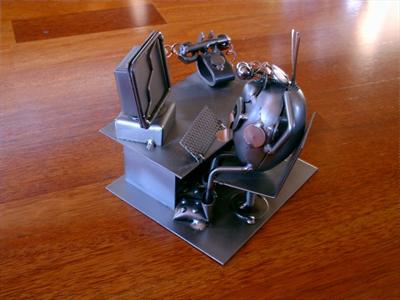

Supplement: S1 Dataset — (ZIP) [file pone.0149328.s001.zip › S1_Dataset/0_15_15644.jpg]

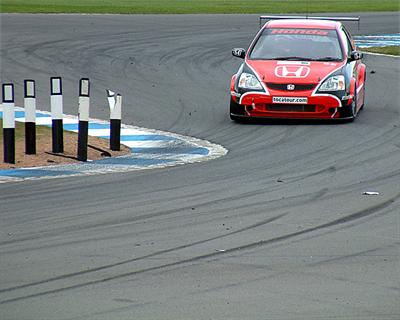

Supplement: S1 Dataset — (ZIP) [file pone.0149328.s001.zip › S1_Dataset/0_15_15742.jpg]

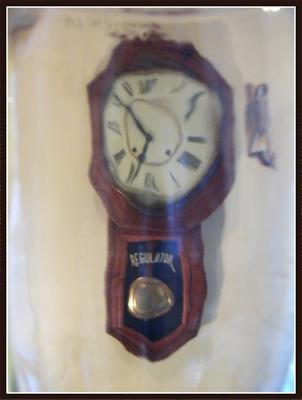

Supplement: S1 Dataset — (ZIP) [file pone.0149328.s001.zip › S1_Dataset/0_15_15859.jpg]

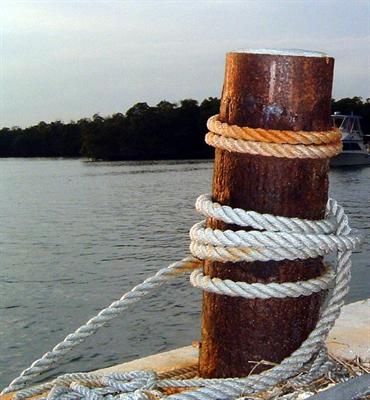

Supplement: S1 Dataset — (ZIP) [file pone.0149328.s001.zip › S1_Dataset/0_15_15935.jpg]

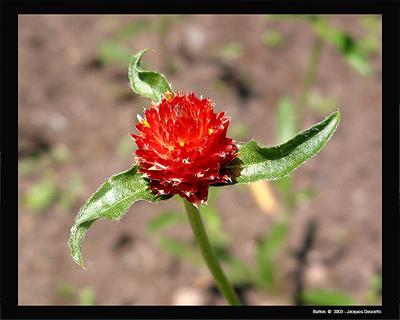

Supplement: S1 Dataset — (ZIP) [file pone.0149328.s001.zip › S1_Dataset/0_16_16030.jpg]

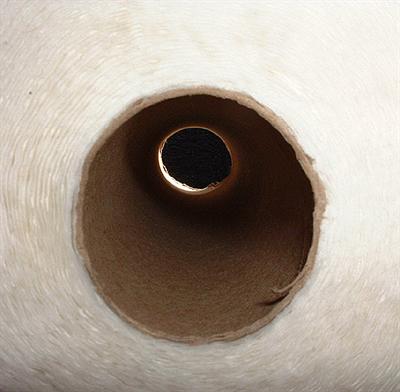

Supplement: S1 Dataset — (ZIP) [file pone.0149328.s001.zip › S1_Dataset/0_16_16079.jpg]

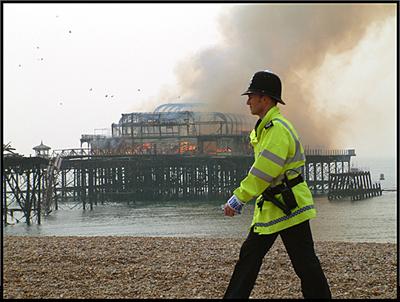

Supplement: S1 Dataset — (ZIP) [file pone.0149328.s001.zip › S1_Dataset/0_16_16431.jpg]

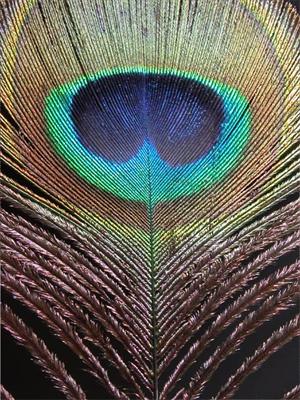

Supplement: S1 Dataset — (ZIP) [file pone.0149328.s001.zip › S1_Dataset/0_16_16283.jpg]

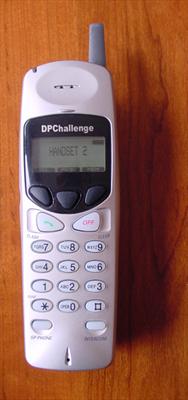

Supplement: S1 Dataset — (ZIP) [file pone.0149328.s001.zip › S1_Dataset/0_16_16905.jpg]

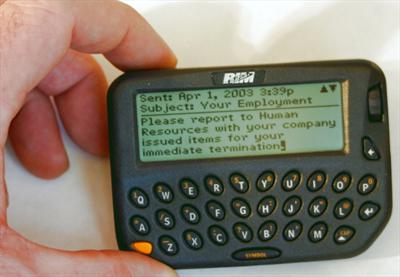

Supplement: S1 Dataset — (ZIP) [file pone.0149328.s001.zip › S1_Dataset/0_16_16940.jpg]

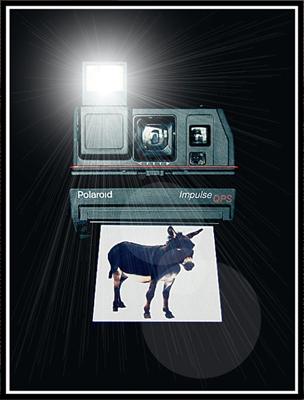

Supplement: S1 Dataset — (ZIP) [file pone.0149328.s001.zip › S1_Dataset/0_16_16947.jpg]

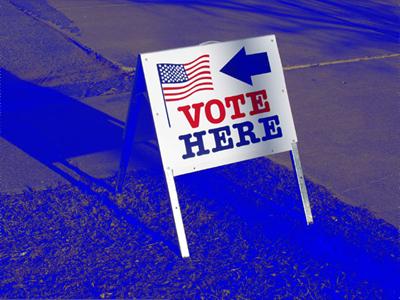

Supplement: S1 Dataset — (ZIP) [file pone.0149328.s001.zip › S1_Dataset/0_16_16968.jpg]

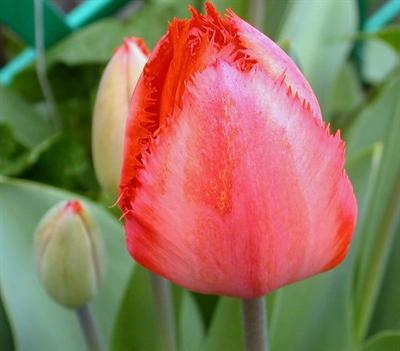

Supplement: S1 Dataset — (ZIP) [file pone.0149328.s001.zip › S1_Dataset/0_17_17022.jpg]

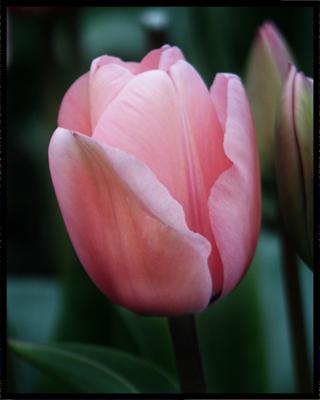

Supplement: S1 Dataset — (ZIP) [file pone.0149328.s001.zip › S1_Dataset/0_17_17057.jpg]

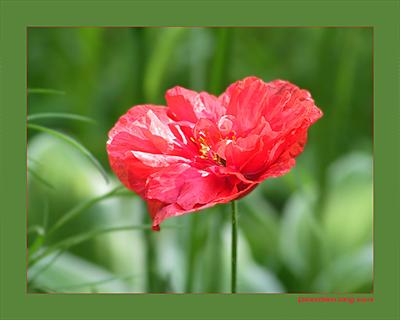

Supplement: S1 Dataset — (ZIP) [file pone.0149328.s001.zip › S1_Dataset/0_17_17251.jpg]

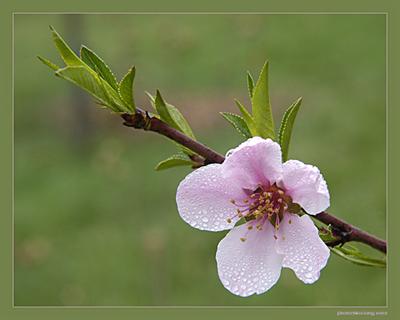

Supplement: S1 Dataset — (ZIP) [file pone.0149328.s001.zip › S1_Dataset/0_17_17275.jpg]

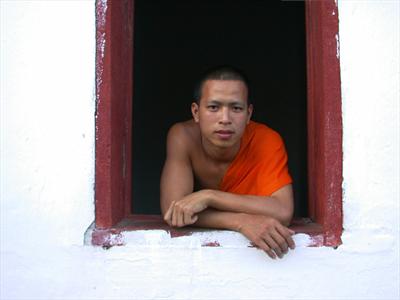

Supplement: S1 Dataset — (ZIP) [file pone.0149328.s001.zip › S1_Dataset/0_17_17350.jpg]

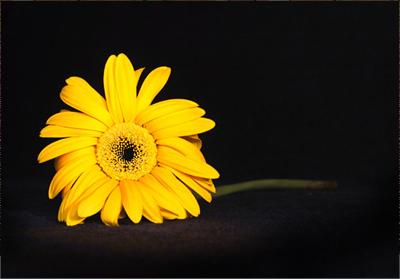

Supplement: S1 Dataset — (ZIP) [file pone.0149328.s001.zip › S1_Dataset/0_17_17369.jpg]

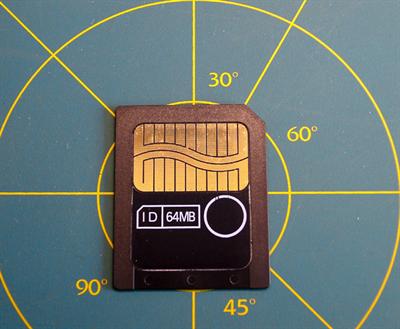

Supplement: S1 Dataset — (ZIP) [file pone.0149328.s001.zip › S1_Dataset/0_17_17388.jpg]

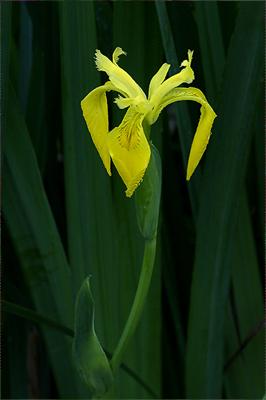

Supplement: S1 Dataset — (ZIP) [file pone.0149328.s001.zip › S1_Dataset/0_17_17525.jpg]

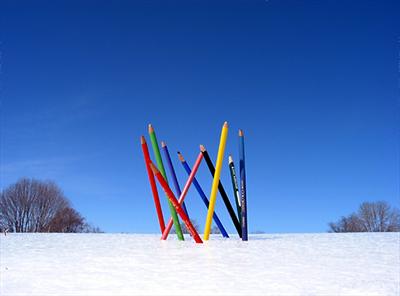

Supplement: S1 Dataset — (ZIP) [file pone.0149328.s001.zip › S1_Dataset/0_17_17526.jpg]

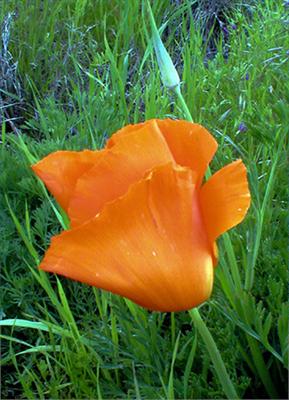

Supplement: S1 Dataset — (ZIP) [file pone.0149328.s001.zip › S1_Dataset/0_17_17553.jpg]

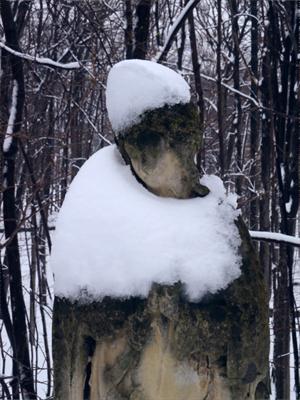

Supplement: S1 Dataset — (ZIP) [file pone.0149328.s001.zip › S1_Dataset/0_17_17674.jpg]

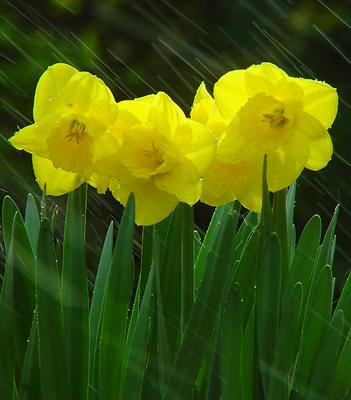

Supplement: S1 Dataset — (ZIP) [file pone.0149328.s001.zip › S1_Dataset/0_18_18160.jpg]

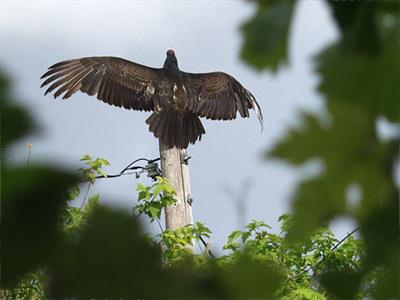

Supplement: S1 Dataset — (ZIP) [file pone.0149328.s001.zip › S1_Dataset/0_18_18219.jpg]

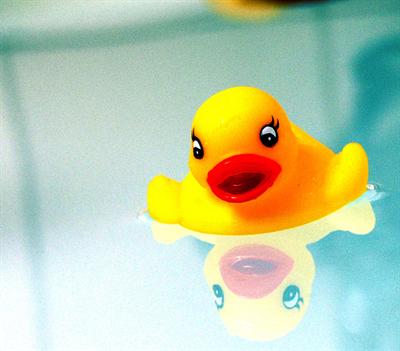

Supplement: S1 Dataset — (ZIP) [file pone.0149328.s001.zip › S1_Dataset/0_18_18310.jpg]

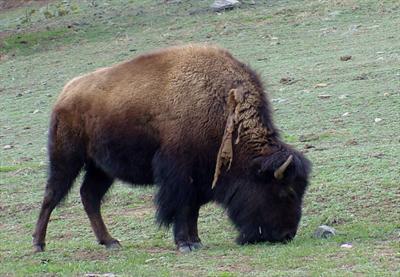

Supplement: S1 Dataset — (ZIP) [file pone.0149328.s001.zip › S1_Dataset/0_18_18562.jpg]

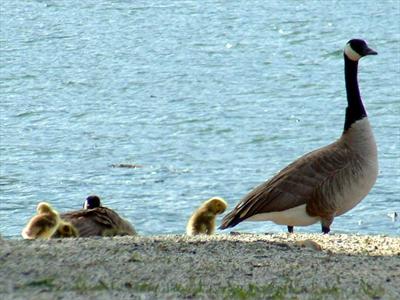

Supplement: S1 Dataset — (ZIP) [file pone.0149328.s001.zip › S1_Dataset/0_18_18636.jpg]

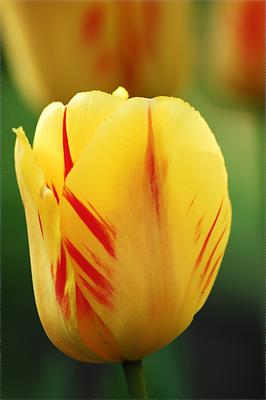

Supplement: S1 Dataset — (ZIP) [file pone.0149328.s001.zip › S1_Dataset/0_18_18653.jpg]

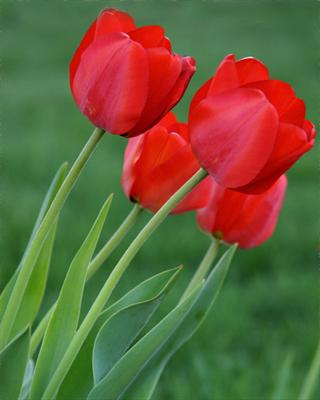

Supplement: S1 Dataset — (ZIP) [file pone.0149328.s001.zip › S1_Dataset/0_18_18720.jpg]

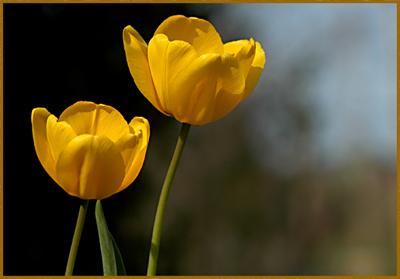

Supplement: S1 Dataset — (ZIP) [file pone.0149328.s001.zip › S1_Dataset/0_18_18723.jpg]
